# Methylated tirilazad may mitigate oligofructose-induced laminitis in horses

**DOI:** 10.3389/fmicb.2024.1391892

**Published:** 2024-09-25

**Authors:** Maimaiti Tuniyazi, Ruibo Tang, Xiaoyu Hu, Naisheng Zhang

**Affiliations:** College of Veterinary Medicine, Jilin University, Changchun, China

**Keywords:** horse, laminitis, methylated tirilazad, matrix metalloproteinases, gut microbiota

## Abstract

Laminitis is a serious health condition that can cause severe pain and lameness in horses. Due to lack of understanding of laminitis, treatments often fail to achieve the desired results. In recent years, we have begun to recognize that laminitis may involve a complex interaction between local and systemic inflammation. Dysbiosis of the gut microbiota has been linked in the development of systemic inflammation, and our previous findings suggest that the development of laminitis is closely linked to the production of harmful metabolites of the gut microbiota. In addition, it was found that localized lesions in the hoof, especially lamellar injuries, are the most direct cause of laminitis. Matrix metalloproteinases have been found to be strongly associated with the development of laminitis. Recent discovery has found that methylated tirilazad has a role in repairing laminar tissue *in vitro*. However, its efficacy in horses never has been studied. Therefore, we aimed to investigate the efficacy of methylated tirilazad (product name: PTP-102) in the prevention/treatment of oligofructose-induced laminitis. The results showed that oligofructose successfully induced laminitis in horses, resulting in detreated clinical signs. Blood indices (including inflammation-related indices and other related indices) were significantly increased. Observations of dissection and staining showed significant bleeding, swelling, and damage to hoof tissue. Analysis of the gut microbiota showed a significant decrease in abundance and diversity, and a significant increase in the relative abundance of specific bacteria. Following methylated tirilazad intervention, there were a significant improvement in clinical signs, blood markers and lamellar tissue damage. Additionally, methylated tirilazad positively influenced the gut microbiota structure by reducing the relative abundance of genera closely associated with the development of equine laminitis. This suggests that some of the therapeutic mechanism of methylated tirilazad may be linked to its effects on the gut microbiota. Notably, methylated tirilazad had better effect in the treatment group than the prophylactic group, indicating the post-diagnosis utility of methylated tirilazad for laminitis management.

## Introduction

Laminitis is a condition that causes severe pain and disability in horses, often leading to significant lameness and distress. For centuries laminitis has been known as an ailment that not results in significant financial losses but also deeply affects horse owners, trainers, veterinarians and farriers emotionally. The complex nature of laminitis with its understanding of causes and often disheartening treatment outcomes has long puzzled those in the horse veterinary community. Laminitis involves a breakdown in attachment between the phalanx and inner hoof wall (Mungall and Pollitt, 1999). Despite ongoing research efforts, there is still a lack of understanding of the precise physiological mechanisms underlying laminitis in horses.

Recent study results suggest that it may involve interactions between inflammation within the hoof and systemic inflammation throughout the horse body (Tuniyazi et al., 2021).

The gut serves as the residence for microbiota that plays an important role in food digestion and supplying necessary nutrients for the horses well-being (Kauter et al., 2019). Microbiota refers to the entire microorganisms including bacteria, fungi, and viruses. Among them, bacteria are the most widely and deeply studied, and has the most significant impact on the host health. Therefore, the normal composition of gut microbiota is vital. However, dietary (Shepherd et al., 2012; Bulmer et al., 2019), environmental (Salem et al., 2018), pathological (Costa et al., 2012; Milinovich et al., 2006), and pharmacological factors (Costa et al., 2015) can lead to gut microbiota dysbiosis in horses. Meanwhile, our previous study suggested that the dysbiosis of the gut microbiota could result in development of laminitis (Tuniyazi et al., 2021). In that study we found that oligofructose induced laminitis disrupted the horse gut microbiota with lowered bacterial diversity and richness as well as increased abundances of bacterial genera such as *Lactobacillus*, *Streptococcus*, and *Megashaera*. In addition, systematic inflammation was followed by these changes with increased production of lipopolysaccharide (LPS), histamine, and lactic acid. Therefore, anti-inflammatory drugs may be a choice for laminitis treatment (Belknap and Black, 2012; Belknap, 2010).

Hoof lamellar damage is considered as the most direct cause of laminitis (Patterson-Kane et al., 2018), and studies have suggested that matrix metalloproteinases (MMPs) may involve in lamellar injury (French and Pollitt, 2004a, 2004b; Kyaw-Tanner et al., 2008; Li et al., 2015). Based on this findings, some studies explored the effects of some drugs such as phosphodiesterase (Fugler et al., 2013), and pentoxifylline (Fugler et al., 2013, 2010), on MMPs inhabitation. However, the studies are few and need further investigation. Recently, Byrock Technologies Ltd. (Ireland) found that methylated tirilazad inhibits MMPs and promotes the repair of damaged laminar tissues in an *in vitro* study. Therefore, we hypothesized that methylated tirilazad may have a preventive/therapeutic effect on laminitis.

The aims of this study was to investigate the therapeutic effect of methylated tirilazad on oligofructose-induced equine laminitis.

## Materials and methods

### Animals and diet management

This research involved 20 healthy Mongolian horses (consisting of 10 male and 10 female, aged 3 years, weighing 308 ± 26 kg) that were purchased by the authors from a stud farm and born during the 2020 foaling season. They have been raised from birth in a constantly regulated environments for meat production. The authors conducted a thorough evaluation of the medical records of the horses, detailing their selection of these previous illnesses, treatments, and veterinary clinical examinations, before finalizing the selection of 20 horses. Only horses with good health records were selected for the study, meaning they showed no signs of digestive problems, had not been recently exposed to antibiotics or deworming medications, and had not experienced long-distance transportation in the past 3 months.

Dietary composition has a strong influence on the gut microbiota of horses (Garber et al., 2020). Although the selected horses were managed under the same feeding conditions and exercise regimens, additional precautions were taken to create a highly controlled feeding environment. Thus, the selected horses were isolated and fed a diet based on local forage for 2 months. Each horse was fed a daily amount of feed equal to 2% of its body weight on a dry matter basis (Dugdale et al., 2010). The horses had unrestricted access to water, and no additional dietary supplements.

To ensure impartiality of the results, the horses were randomly assigned to four different groups: control (control; *n* = 5), laminitis (laminitis + no treatment; *n* = 5), prophylactic (methylated tirilazad + laminitis; *n* = 5), and treatment (laminitis + methylated tirilazad; *n* = 5). Randomization was performed using a computer-generated randomization sequence.^1^ The researchers involved in the data collection and analysis were blinded to the group assignments where possible.

Each horse was housed individually to prevent any potential cross-contamination and to maintain isolation between the groups for the duration of the study period.

### Induction of laminitis

**Figure 1:**
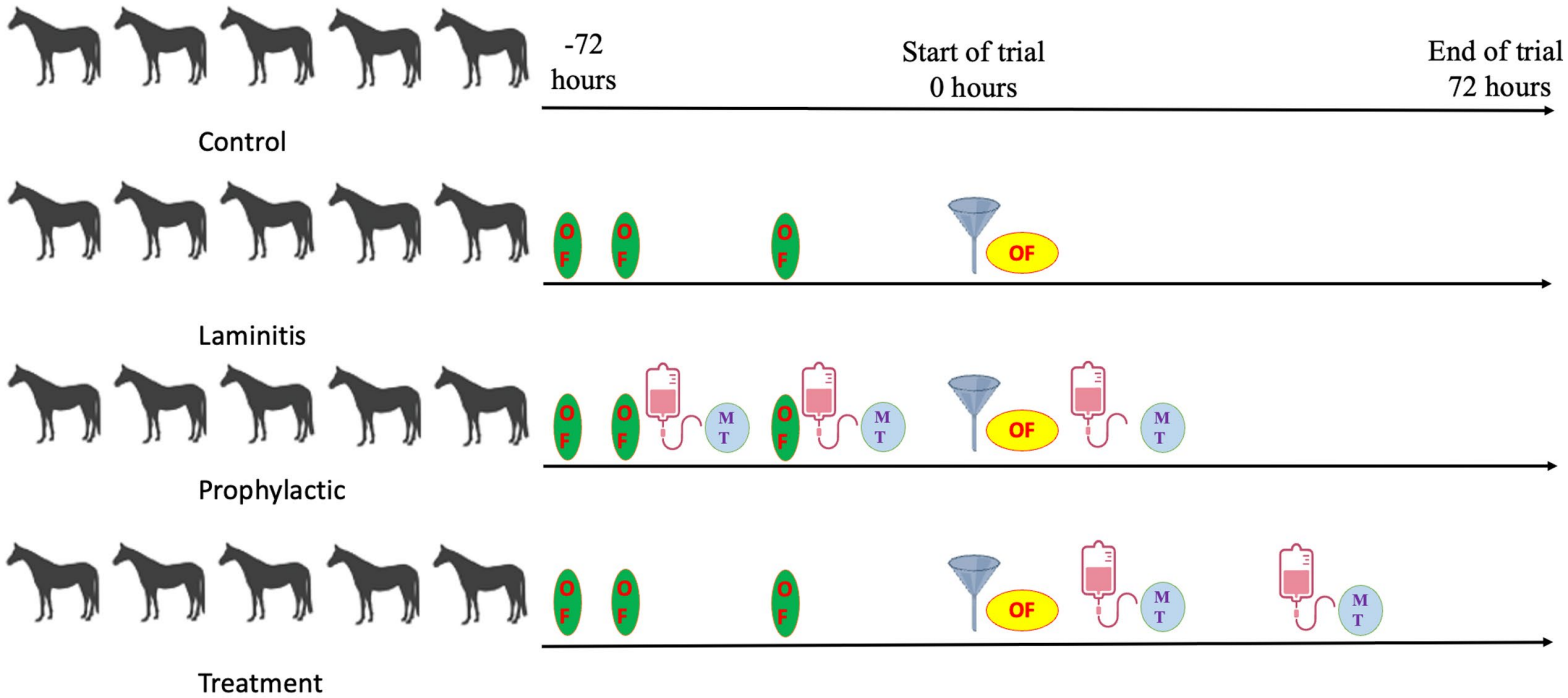
Study timeline overview.

We successfully induced laminitis in horses experimentally using according to previous studies (Tuniyazi et al., 2021; Milinovich et al., 2006; van Eps and Pollitt, 2006). After acclimating the horses to the specified diet and individual housing conditions, each horse received different intervention. In the control group, no interventions were performed during the study phase. In the laminitis group, each horse within this group was given a 10 g/kg body weight of oligofructose (dissolved in 10 L warm water) with a nasogastric tube. Adjunctive treatment was provided as required and there was no experimental treatment. Horses with an Obel score of 3 were humanely euthanized. In the prophylactic group, horses were treated with methylated tirilazad intravenously for 2 days (0 h) prior to the start of the study and were re-treated with methylated tirilazad at the same time as the administration of oligofructose by a nasogastric tube (10 g/kg body weight). In the treatment group, horses received methylated tirilazad IV therapy as the first signs of pyrexia and/or diarrhea and were confirmed to have observable signs of laminitis (Obel score >1). In addition, a second dose of methylated tirilazad was administered 12 h after the first dose. Horses in both the prophylactic and treatment groups were euthanized 72 h after oligofructose administration (Figure 1).

### Sample size determination

The sample size was determined based on previous studies with a similar design (Tuniyazi et al., 2024) as well as power analysis using G*Power software (version 3.1.9.6; Heinrich-Heine-Universität Düsseldorf, Düsseldorf, Germany) (Kang, 2021; Faul et al., 2009). With an expected effect size of 0.95, *α* = 0.4, and power (1 − *β*) = 0.95, a sample size of 5 horses per group was calculated.

### Preparation and dosing of methylated tirilazad

Methylated tirilazad (official name: 21-[4-(2,6-di-1-pyrrolidinyl-4-pyrimidinyl)-1-piperazinyl]-pregna-1,4,9(11)-triene-3,20-dione maleate salt; CAS Number: 153190-29-5; purity: ≥99%) is a compound widely used in biomedical research. Byrock Technologies has determined that this synthetic compound has anti-inflammatory and immunomodulatory effects on equine laminitis through *in vitro* studies.

Thirty-five grams of methylated tirilazad (product name: PTP-102) was produced from Viva Biotechnology (Shanghai, China). In our study, each horse in the prophylactic and treatment groups was injected with an equal amount methylated tirilazad (1.4 g per horse per injection) via the jugular vein. The dosage of 1.4 g methylated tirilazad per horse per injection was determined based on preliminary *in vitro* studies conducted by Byrock Technologies, and scaled for *in vivo* use according to standard pharmacokinetic principles. The dosage was chosen to achieve the predicted therapeutic concentrations while minimizing potential side effects.

The preparation of methylated tirilazad for intravenous injection is shown in Supplementary Figure S1. Figure 1 illustrates a brief timeline of the study and the differences in the timing of methylated tirilazad administration between the two groups.

In addition to the treatment group, we included a prophylactic group to investigate the potential of methylated tirilazad as a treatment and as a preventive effect for laminitis. By evaluating the efficacy of methylated tirilazad in prophylactic therapy, we aimed to provide recommendations for the use of methylated tirilazad in athlete horses prior to high-intensity training or racing, which may increase the risk of developing laminitis. Therefore, by including the prophylactic group, we can explore the preventive efficacy and potential impact of methylated tirilazad in the treatment of equine laminitis.

### Clinical assessments

Throughout the course of the study, a series of clinical assessments were conducted to evaluate various parameters related to the health and condition of horses. These assessments included measurements of body temperature, fecal pH, lameness score, and diarrhea score. Body temperature was measured using a rectal thermometer, while fecal pH was assessed using a pH meter (KA-118, OEM, Ningbo China). The lameness score was based on the well-established Obel lameness scoring system (Obel, 1948), which considers gait abnormalities, limb loading, and observable signs of pain or discomfort exhibited by the horses during movement. The diarrhea scoring was based on a previously developed scale (McKinney et al., 2020), and the severity of diarrhea was assessed on a scale ranging from 0 to 5. The scoring system includes the following classifications: 0—normal; 1—soft-formed; 2—pudding-consistency with shape; 3—pudding-consistency that spreads; 4—watery manure with some formed pieces; 5—watery manure without shaped clumps.

Clinical assessments were performed every 4 h after oligofructose administration or at equivalent time points in the control group. However, to better assess the changes among the different groups, clinical assessments were performed using data collected after 72 h of oligofructose administration.

### Serum evaluation

#### Detection of LPS concentration

Blood samples were collected and centrifugated at 3,000 g for 30 min at 4°C. After centrifugation, the supernatant was transferred to a sterile denitrifying glass tube. The LPS concentration was determined using a chromogenic endpoint assay according to the manufacturer’s instructions (MLBIO Biotechnology Co. Ltd., Shanghai, China). The assay has a minimum detection threshold of 0.01 EU/mL for accurate measurement of LPS concentration.

#### Detection of lactic acid, histamine, MMP-1, MMP-2, and MMP-9 concentrations

Blood samples were collected from different groups of horses and centrifuged at 3,000 g for 30 min at 4°C. Serum was collected and measured using an assay. Serum was collected and assayed for lactic acid, histamine, MMP-1, MMP-2, and MMP-9 using a kits following the manufacturer’s instructions (MLBIO Biotechnology Co. Ltd.).

Blood samples for serum evaluation were collected every 8 h after oligofructose administration, or at equivalent time points in the control group. However, to better compare the changes among the different groups, blood serum evaluations were performed using data collected after 72 h of oligofructose administration.

#### Euthanasia

Humane euthanasia was performed on the horses for the necessary pathological evaluations. Euthanasia was performed according to an established protocol using a 1:5 [0.1 mL/kg (Tuniyazi et al., 2021)] xylazine-ketamine composition (IS Abundant Pharmaceutical Co., Ltd., Lanzhou China). Pre-euthanasia drug was injected intravenously through the jugular vein at a rate of 0.5–1 mL/s. Sodium pentobarbital (Feilong Pharmaceutical CO., LTD, Heilongjiang China) was injected through the jugular vein at a dosage of 0.1 mL/kg. To ensure the effectiveness of euthanasia, veterinary experts confirmed that animals were unconsciousness and insensitivity to pain. This confirmation included a needle pinprick test on the surface of the ears to ensure that there were no responses indicating pain or consciousness.

#### Hoof dissection evaluation

Hoof assessment involved a dissection with the goal of examining and analyzing the internal structures. This process included examining various anatomical components, such as the lamellae and hoof wall to identify any signs of pathology or abnormalities. The results of the observations were recorded throughout the dissection and detailed photographs were taken to document any noteworthy findings.

#### Hematoxylin and eosin (H&E) and periodic acid-Schiff (PAS) staining

Within 1 h of euthanasia, hoof tissues were immediately collected and carefully sectioned with a band saw to ensure consistency. Each section measured 10 × 10 × 0.5 mm and included the hoof wall and lamina propria tissue. For consistency, these sections were further divided into 55-mm-square blocks and then fixed in a 4% formalin solution for a period of 24 to 72 h. This standardized fixation process was used to ensure optimal preservation of lamellar tissue in all specimens. Subsequently, fixed blocks of lamellar tissue were embedded in paraffin, according to routine laboratory procedures. To ensure accuracy and consistency, tissue blocks from the same location within the laminar tissue of each horse were collected (Al-Agele et al., 2019). For assessment of lamellar lesions and morphometric analysis, sections were stained according to established H&E and PAS staining protocols as described in previous studies (Karikoski et al., 2015). After staining, the sections were examined under a light microscope using an image capture software and a camera (Olympus, Japan).

#### Total bacterial DNA extraction and Illumina NovaSeq sequencing

Fecal samples from 20 horses (5 per group) were used for bacterial DNA extraction and subsequent microbial analysis. DNA extraction from equine fecal samples was carried out using the CTAB method as per the manufacturer’s protocol. The CTAB method effectively enables the recovery of DNA from trace amounts of the sample and has been validated for the preparation of DNA from diverse bacterial species (Mavrodiev et al., 2021). Blank samples were treated with nuclear-free water. The extracted total DNA was eluted in 50 μL of Elution buffer and stored at −80°C until further analysis.

To amplify the V3–V4 region of the 16S rDNA genes, we employed a primer set (314F and 805R) with barcodes attached to the 5′ ends of the primers. The PCR amplification reactions were performed in a total volume of 25 μL, containing 25 ng of template DNA, 12.5 μL of PCR Premix, and PCR-grade water for volume adjustment. The PCR conditions for amplifying prokaryotic 16S fragments involved an initial denaturation at 98°C for 30 s, followed by 32 cycles of denaturation at 98°C for 10 s, annealing at 54°C for 30 s, extension at 72°C for 45 s, and a final extension at 72°C for 10 min. The amplification products were confirmed using 2% agarose gel electrophoresis. To remove PCR inhibitors and purify the amplified products, AMPure XT beads were used. The quantity and quality of the amplicon libraries were assessed on Agilent 2100 Bioanalyzer and the Library Quantification Kit for Illumina, respectively. The libraries were sequenced on a NovaSeq PE250 platform, producing paired-end reads. The sequences were quality filtered using filter conditions specified in fqtrim (v0.94) to obtain high-quality clean tags. Vsearch software (v2.3.4) was employed to remove chimeric sequences, and sequences were randomly normalized using DADA2 software to assess alpha diversity and beta diversity. For normalization of feature abundance, the SILVA database classifier was employed and scaled based on the relative abundance of each sample.

The microbial structure in different groups of equine fecal samples was analyzed using different methods, such as observed species, Chao1, Shannon, Simpson, and Godds_coverage, Pielou_e, as well as principal component analysis (PCA), principal coordinate analysis (PCoA), UPGMA clustering, and non-metric multidimensional scaling (NMDS). To determine the effect size, linear discriminant analysis effect size (LEfSe) was used to identify bacterial taxa with differential abundance between the groups (Segata et al., 2011). Furthermore, Pearson correlation analysis was performed to investigate the relationship between fecal microbiota and host parameters using a web-based system developed by LC-Bio Technology.^2^ Lastly, Phylogenetic Investigation of Communities by Reconstruction of Unobserved States (PICRUSt2) analysis was performed to identify bacterial functions across the different groups (Douglas et al., 2020).

### Statistical analysis

All statistical analyses were conducted using GraphPad Prism software (Version 10.0.3, GraphPad Software, Inc., San Diego, CA). Data are expressed as mean values ± standard error of the mean (SEM). All data were tested for normality using the Shapiro–Wilk test. For normally distributed data, differences between groups were determined by two-way ANOVA followed by post-hoc Tukey tests. For non-normally distributed data, differences were determined using the Kruskal–Wallis test followed by post-hoc Dunn tests. Alpha diversity was calculated using the Kruskal–Wallis test. As noted above, the correlations between microbiota composition and clinical parameters were assessed using Pearson correlation analyses. A *p* < 0.05 is considered to be statistically significant. Statistical significance was denoted by ^*^p < 0.05, ^**^p < 0.01, ^***^p < 0.001, and ^****^p < 0.0001.

## Results

### Horse clinical features in different groups

**Figure 2:**
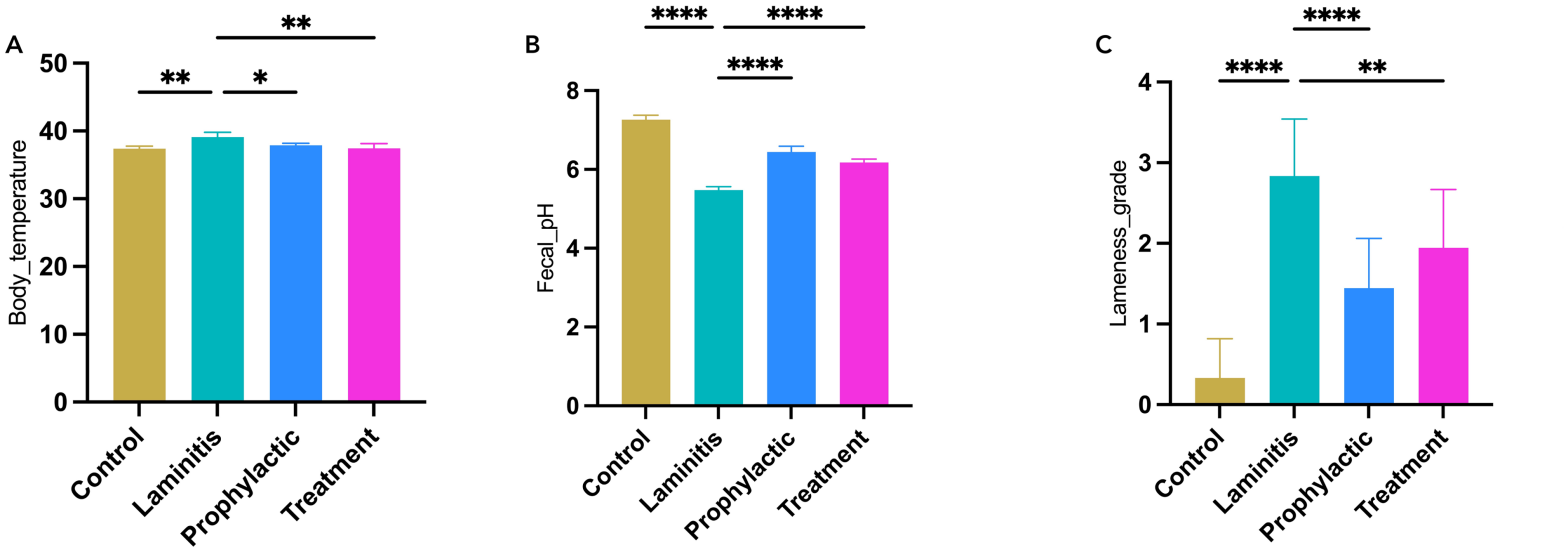
Clinical parameters of horses in different groups. **(A)** Body temperature. **(B)** Fecal pH. **(C)** Lameness score.

**Figure 3:**
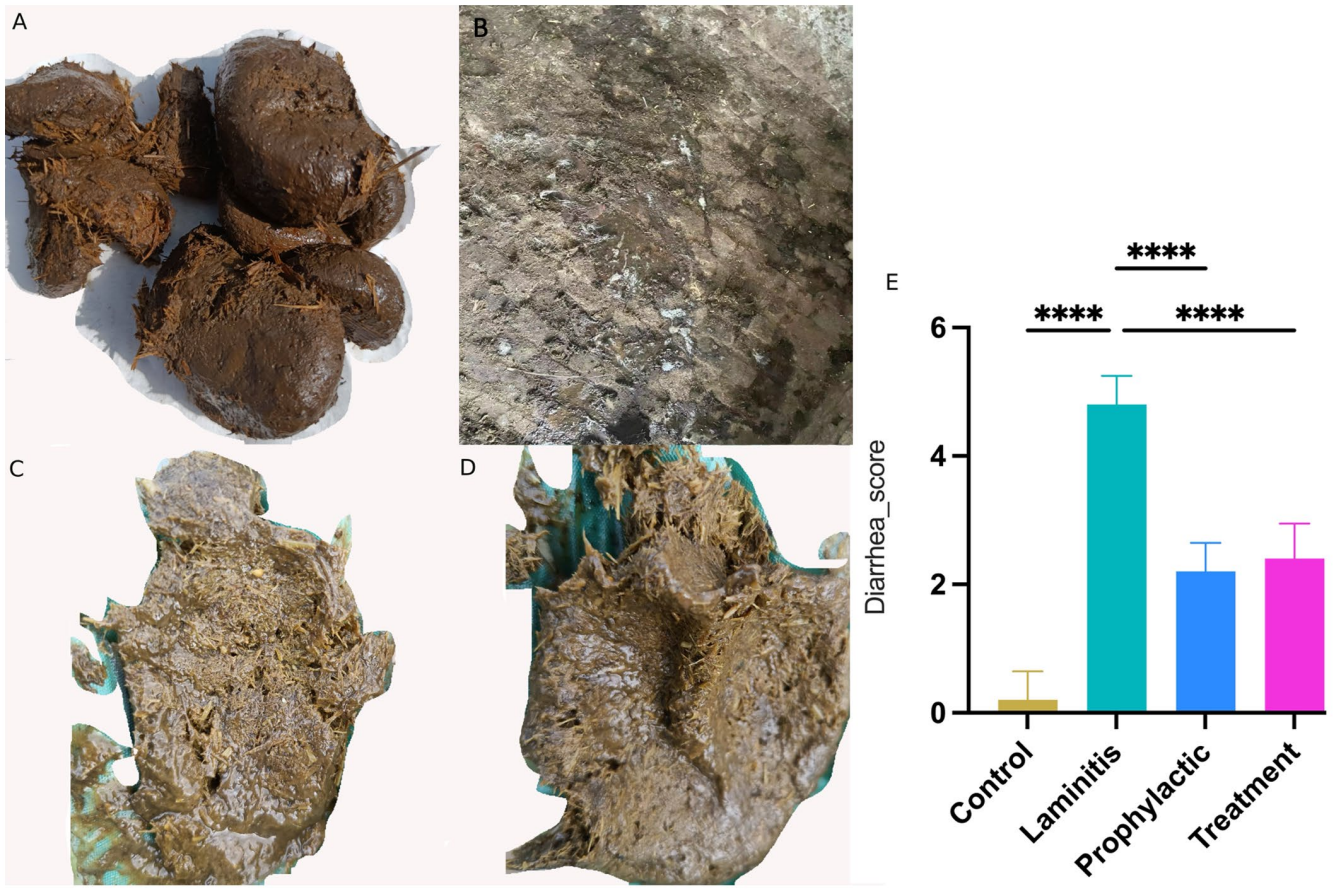
Fecal appearance and diarrhea score in different groups. **(A)** Stool of control group horse. **(B)** Stool of laminitis group horse. **(C)** Stool of prophylactic group horse. **(D)** Stool of treatment group horse. **(E)** Diarrhea score in different groups.

To comprehensively explore the clinical characteristics of different groups, our study provided an in-depth analysis of various parameters, including body temperature, fecal pH, lameness score (Figure 2), and fecal consistency and diarrhea score (Figure 3). Specifically, horses in the laminitis group had significantly higher body temperatures than those in the control group (*p* = 0.0011). After receiving methylated tirilazad, horses in both the prophylactic and treatment groups showed a significant decrease in body temperature compared with the laminitis group (laminitis vs. prophylactic, *p* = 0.016; laminitis vs. treatment, *p* = 0.0014). Fecal pH was significantly lower in horses in the laminitis group than in those in the control group (*p* < 0.0001). After methylated tirilazad intervention, there was a clear increase in fecal pH in both the prophylactic and treatment groups compared to that in the laminitis group (*p* < 0.0001; *p* < 0.0001). In addition, lameness scores were significantly higher in horses in the laminitis group than in those in the control group (*p* < 0.0001). After methylated tirilazad, the lameness scores were significantly lower in both the prophylactic and treatment groups than in the laminitis group (*p* < 0.0001; *p* = 0.0024).

Notably, horses in the laminitis group exhibited watery diarrhea in their feces compared to the control group. Following the intervention, both the prophylactic and treatment groups achieved significant efficacy in alleviating diarrhea symptoms, as evidenced by improvement in fecal appearance and diarrhea scores compared to the laminitis group. In terms of diarrhea scores, horses in the laminitis group had significantly higher scores than those in the control group (*p* < 0.0001). After receiving methylated tirilazad, the diarrhea scores of the horses in both the prophylactic and treatment groups were significantly lower than those of the horses in the laminitis group (*p* < 0.0001; *p* < 0.0001).

### Serum assessments in in different groups of horses

**Figure 4:**
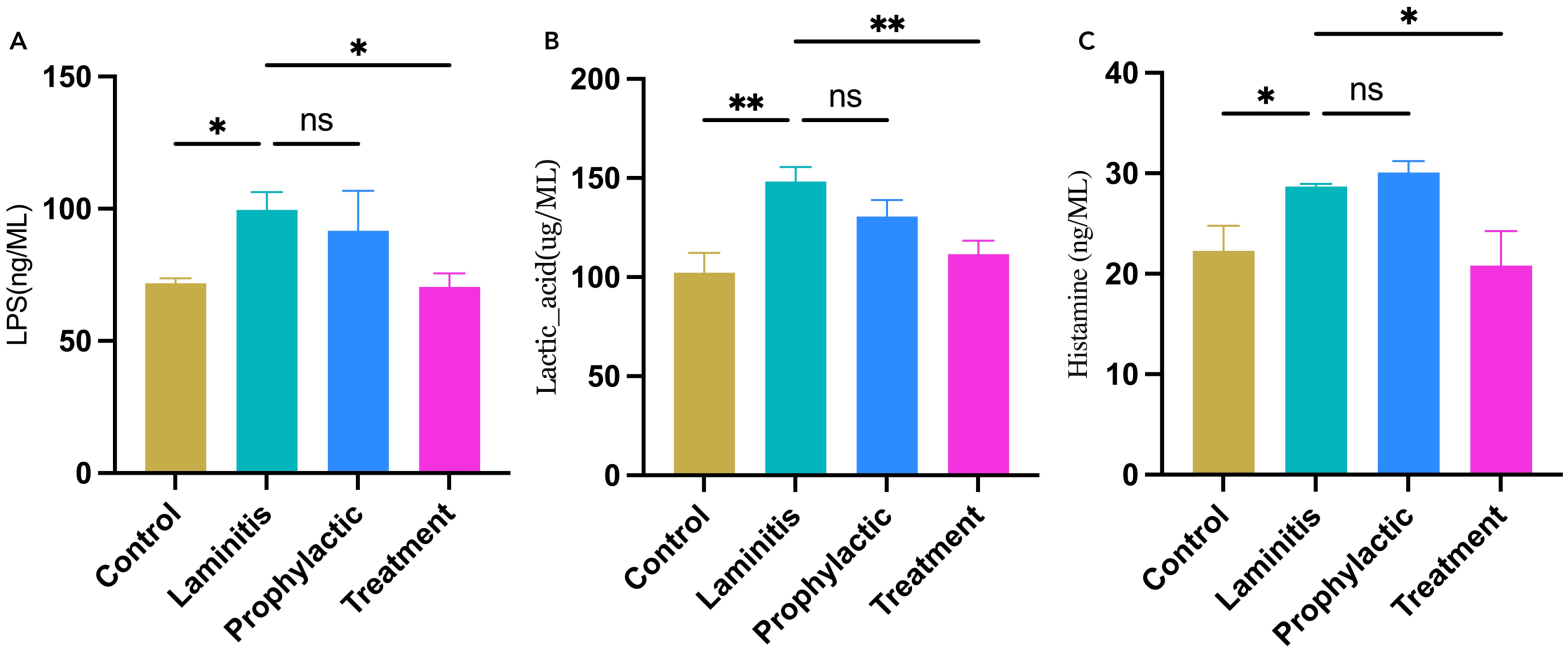
Blood serum concentrations of inflammatory cytokines in different groups. **(A)** LPS. **(B)**. Lactic acid. **(C)** Histamine.

To assess the systemic inflammatory response in the study population, we analyzed the serum levels in the different groups of horses, as shown in Figure 4. Specifically, LPS concentrations were significantly higher in the laminitis group than in the control group (*p* = 0.0315). After receiving methylated tirilazad, there was no significant change in the prophylactic group (*p* = 0.7012), whereas the treatment group showed a significant decrease (*p* = 0.0253) compared to the laminitis group. In addition, the lactate concentration was significantly higher in horses in the laminitis group than in those in the control group (*p* = 0.0029). After receiving methylated tirilazad, the prophylactic group did not show any significant change (*p* = 0.1667), whereas the treatment group showed a significant decrease (*p* = 0.0091) compared to the laminitis group. In addition, histamine concentrations were significantly higher in horses in the laminitis group than in those in the control group (*p* = 0.0498). No significant changes were observed in the prophylactic group after methylated tirilazad administration (*p* = 0.8726), whereas a significant decrease was observed in the treated group compared to that in the laminitis group (*p* = 0.0203).

**Figure 5:**
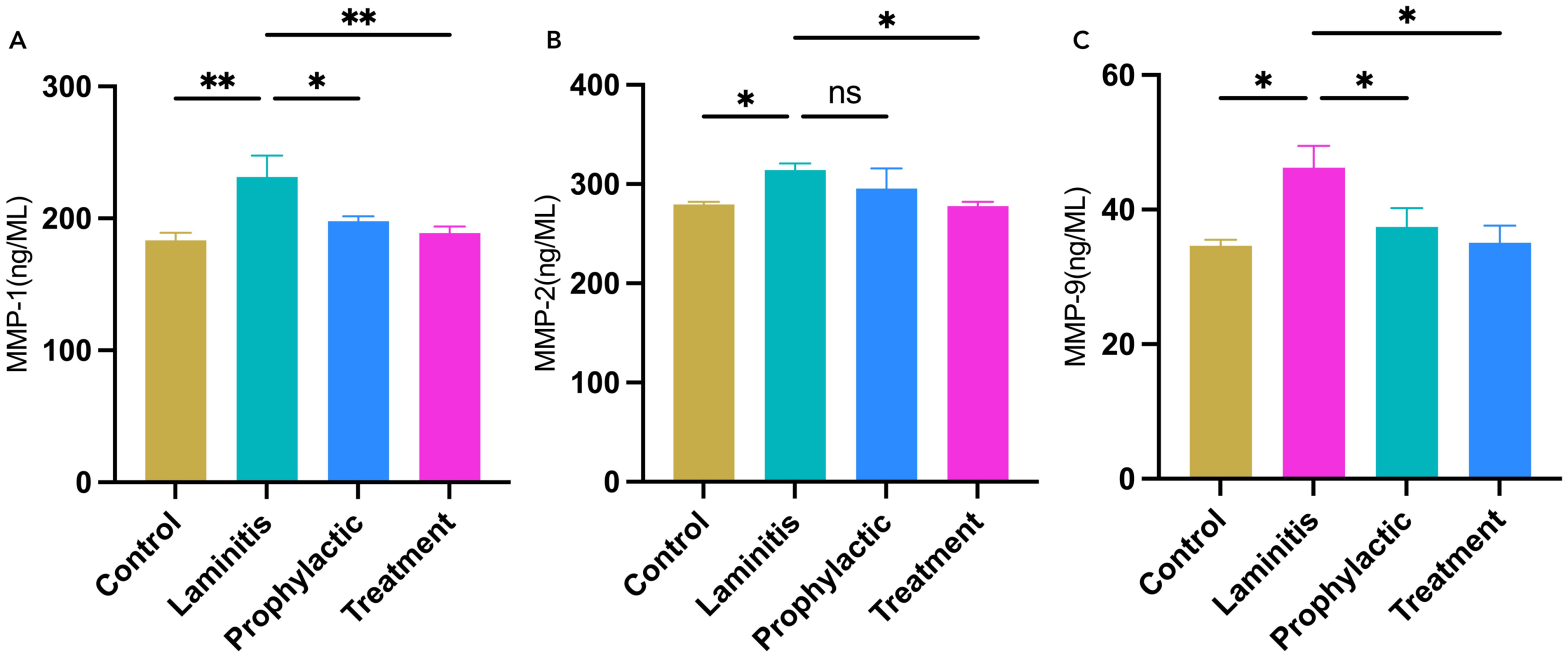
Blood serum concentrations of MMPs in different groups. **(A)** MMP-1. **(B)** MMP-2. **(C)** MMP-9.

Subsequently, we assessed changes in the serum levels of MMPs in the different equine groups, as shown in Figure 5. Notably, MMP-1 concentrations were significantly higher in the laminitis group than in the control group (*p* = 0.0025). Methylated tirilazad intervention resulted in a decrease in MMP-1 concentrations in both the prophylactic and treatment groups compared with the laminitis group (*p* = 0.0152; *p* = 0.0047). In addition, MMP-2 concentrations were significantly higher in the laminitis group than in the control group (*p* = 0.0224). After receiving methylated tirilazad, no significant changes were observed in the prophylactic group (*p* = 0.2115), whereas a significant decrease was observed in the treatment group compared with the laminitis group (*p* = 0.0182). In addition, MMP-9 concentration was significantly higher in horses in the laminitis group than in those in the control group (*p* = 0.0105). After methylated tirilazad administration, MMP-9 concentrations were significantly lower in both the prophylactic and treatment groups than in the laminitis group (*p* = 0.0365 and *p* = 0.0127, respectively).

### Anatomical assessment of hooves

**Figure 6:**
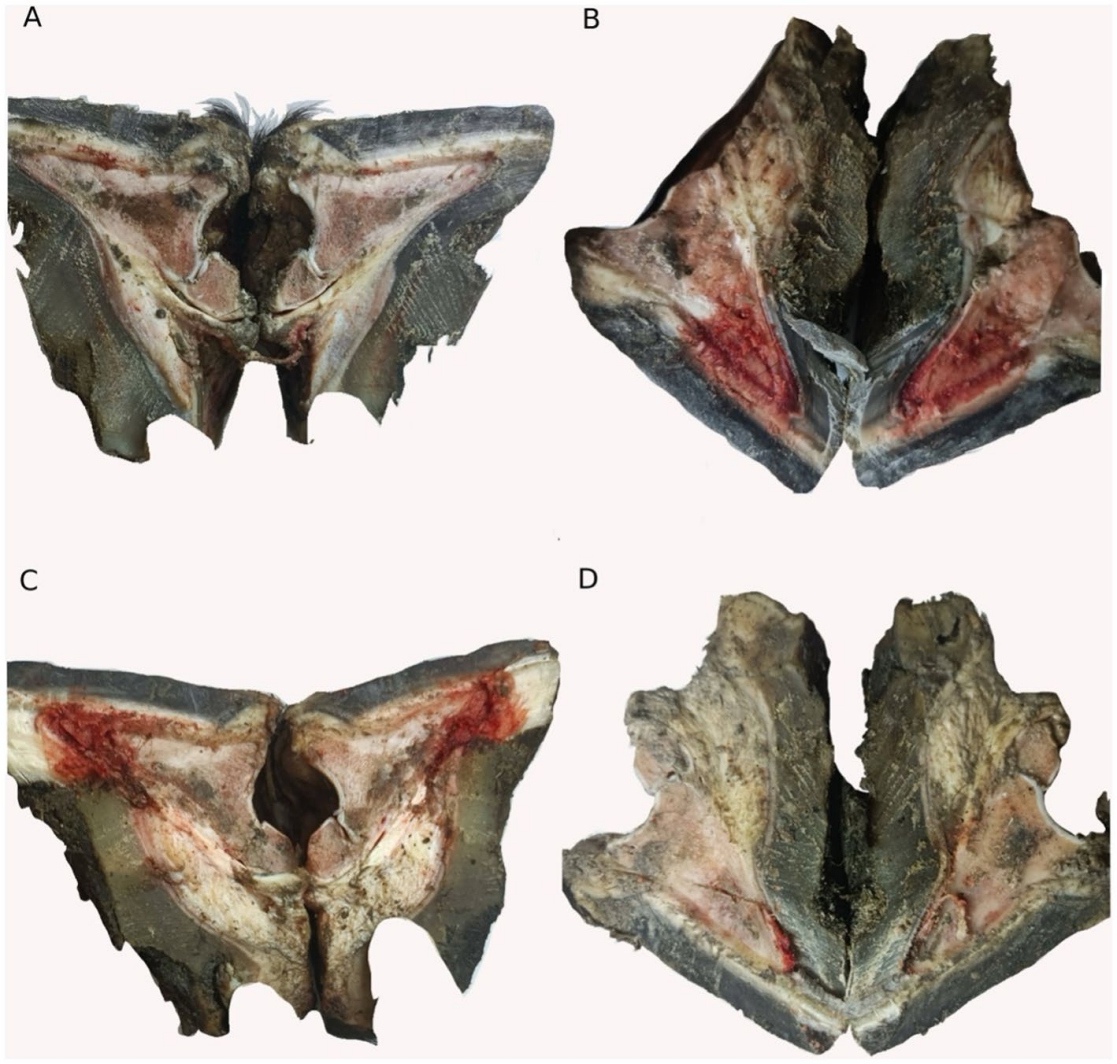
Hoof dissection observation of different groups. **(A)** Control group. **(B)** Laminitis group. **(C)** Prophylactic group. **(D)** Treatment group.

In this study, we performed an anatomical examination of the hooves of the different groups, as shown in Figure 6. The hooves of the laminitis group showed obvious signs of hemorrhage and tissue swelling compared to those of the control group (Figures 6A,B). However, after treatment with methylated tirilazad, both the prophylactic (Figure 6C) and treatment groups (Figure 6D) showed significant improvement, especially a reduction in hemorrhage. Importantly, the results in the treatment group were particularly favorable and similar to those in the control group.

### Histopathological assessment of lamellar tissue in different groups

**Figure 7:**
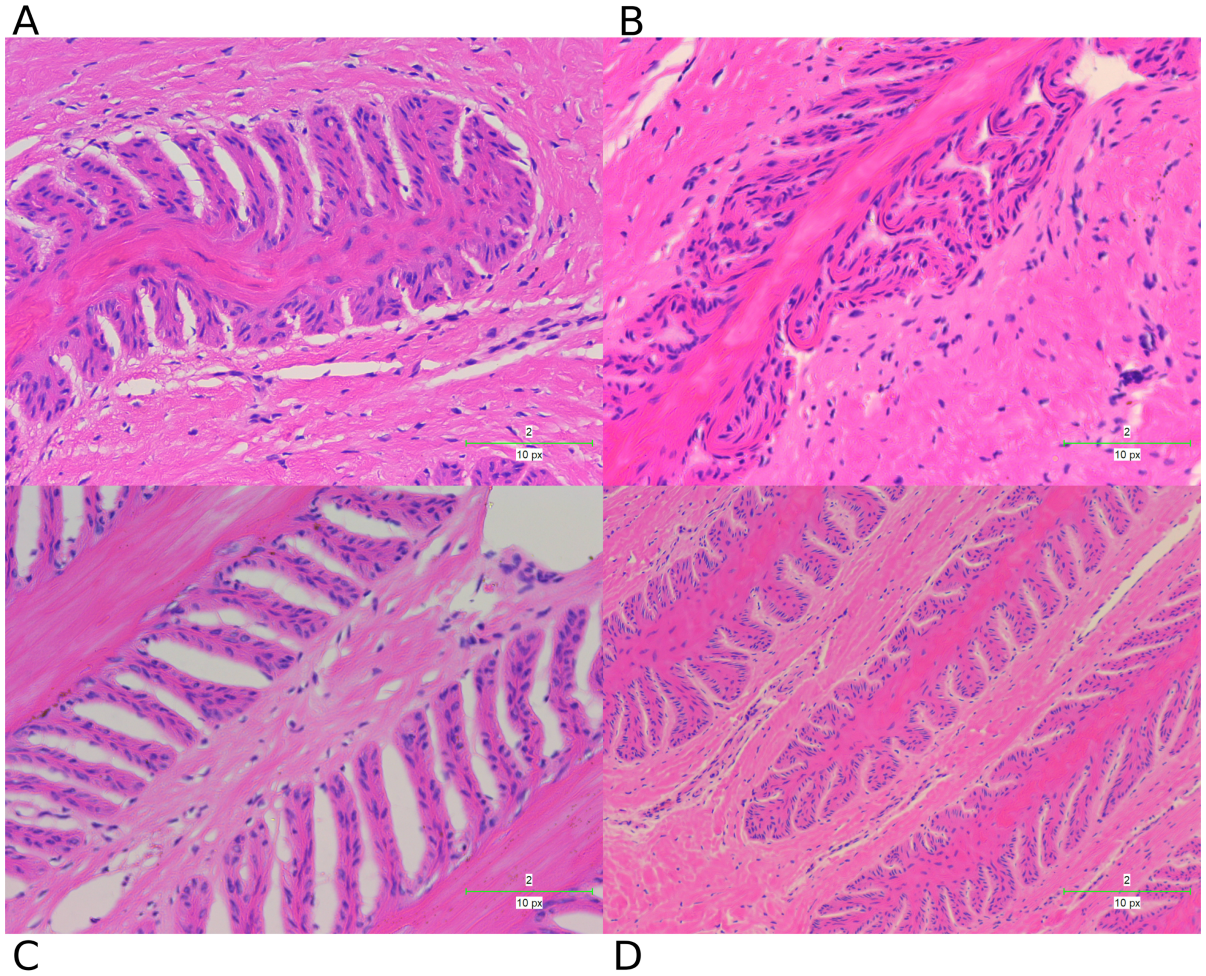
H&E staining examination of hoof tissues in different groups. **(A)** Control group. **(B)** Laminitis group. **(C)** Prophylactic group. **(D)** Treatment group.

**Figure 8:**
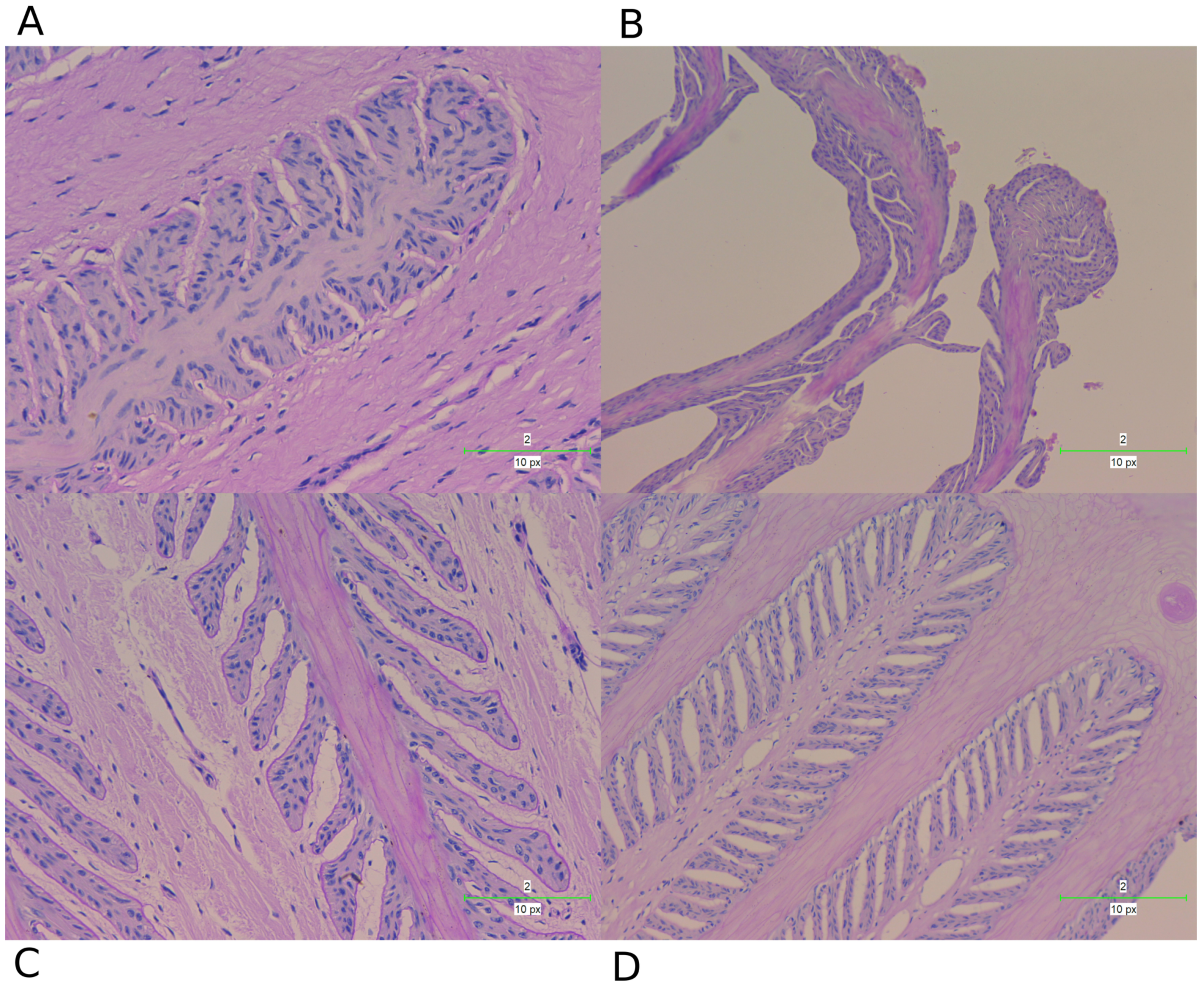
PAS staining examination of hoof tissues in different groups. **(A)** Control group. **(B)** Laminitis group. **(C)** Prophylactic group. **(D)** Treatment group.

To further investigate the changes in the lamellar tissue of horses receiving various interventions, we performed a histopathological assessment using H&E (Figure 7) and PAS (Figure 8) staining.

These staining techniques are effective in delineating the separation between the epidermal and dermal layers of the hoof, a key feature of laminitis. Notably, the laminitis group exhibited significant hoof lamella separation compared with the control group (Figures 7A,B, 8A,B).

Histological examination revealed the presence of inflammatory cells including neutrophils and macrophages in the laminitis group. In addition, irregularities, distortions, and tissue breakdown are evident in the epidermal tissue (hoof wall). Following treatment with methylated tirilazad, both the prophylactic (Figures 7C, 8C) and treatment groups (Figures 7D, 8D) showed significant improvement in repairing the damaged laminar tissue structure. In addition, the treatment group showed better results, similar to the histological features observed in the control group.

### Assessment of fecal microbiota profiles

To investigate the relationship between the composition of gut microbiota and various health conditions in horses, fecal microbiota analysis was performed. The results related to α-diversity (Supplementary Figure S2) showed a significant decrease in the observed species, Shannon, Simpson, Chao1, Goods coverage, and Pielou_e in the laminitis group compared to those in the control group (*p* < 0.0001, *p* < 0.0001, *p* < 0.0001, *p* = 0.0016, *p* < 0.0001, *p* < 0.0001, respectively). After administration of methylated tirilazad, Shannon, Simpson, and Pielou_e were significantly increased in the prophylactic group compared to those in the laminitis group (*p* = 0.0013; *p* = 0.0013; *p* = 0.0010), whereas there were no significant changes in the observed species, Chao1, and Goods coverage (*p* = 0.0920; *p* = 0.6076; *p* = 0.0977). After methylated tirilazad administration, there was a significant increase in the number of observed species, Shannon, Simpson, Goods coverage, and Pielou_e in the treatment group compared to the laminitis group (*p* = 0.0174; *p* < 0.0001; *p* < 0.0001; *p* = 0.0187; *p* < 0.0001), whereas there was no significant change in Chao1 (*p* = 0.1665).

Beta diversity analysis (Supplementary Figure S3) showed that PCA (R = 0.5794, *p* = 0.001), PCoA (R = 0.6173, *p* = 0.001), UPGMA clustering, and NMDS plots based on unweighted UniFrac distances showed a significant separation between the control and laminitis groups, indicating that the two groups had different microbial compositions. The composition of the gut microbiota changed after the administration of methylated tirilazad (prophylactic and treatment groups). However, the changes were more similar to those observed in the laminitis group than in the control group. More specifically, the UPGMA clustering plot showed no significant differences among the laminitis, prophylactic, and treatment groups. The treatment group was slightly similar to the control group, whereas the prophylactic group was more similar to the laminitis group. These results suggest that methylated tirilazad may have a limited effect on the horse gut microbiota.

### Composition of the gut microbiota at the phylum level

**Figure 9:**
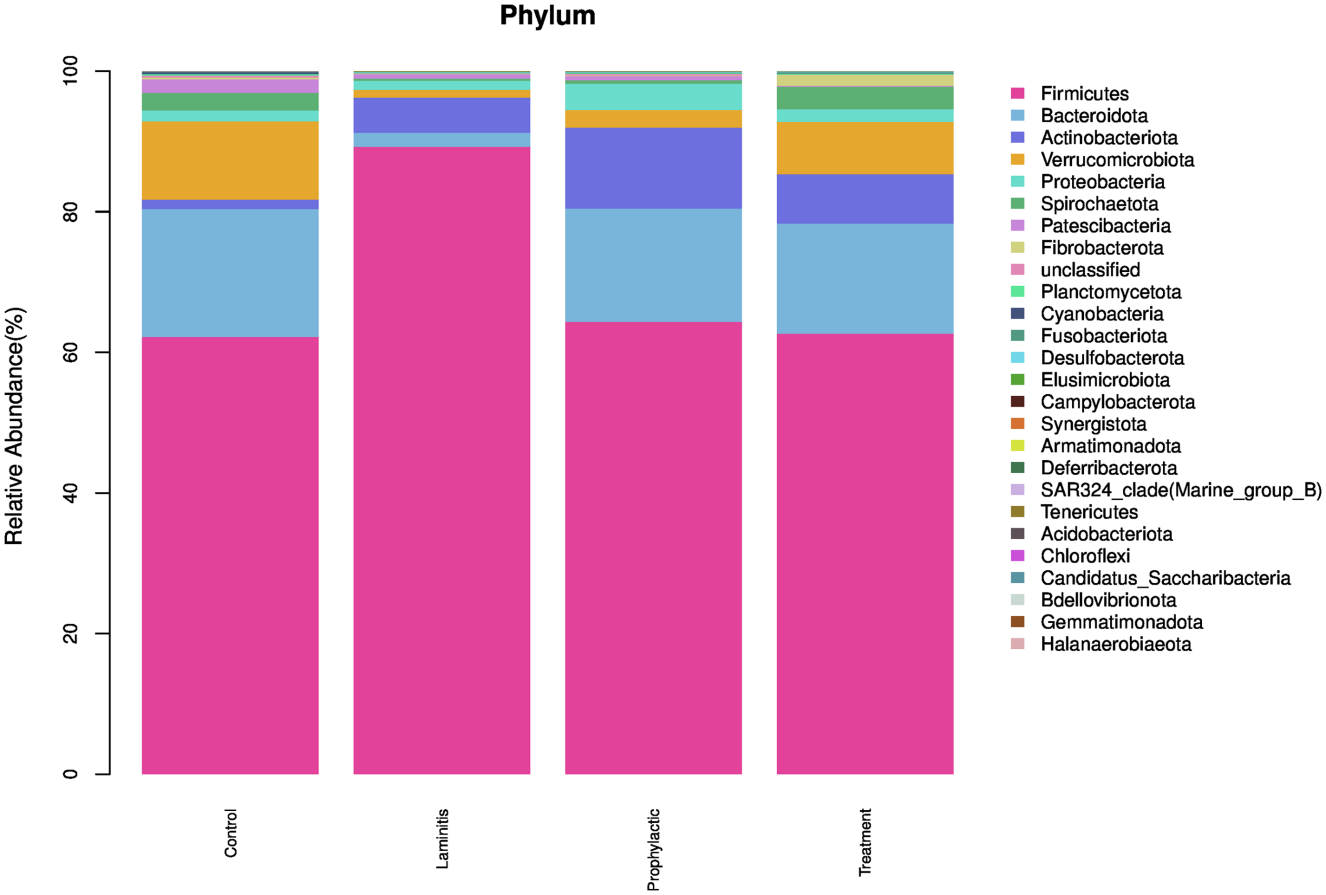
The fecal microbiota compositions in different groups at phylum level.

In order to understand the composition of the fecal microbiota, we analyzed the top 5 phyla in different groups (Figure 9).

In the control group, the dominate (>1%) phylum were *Firmicutes* (62.18%), *Bacteroidota* (18.21%), *Verrucomicrobiota* (11.14%), *Spirochaetota* (2.51%), and *Patescibacteria* (1.91%). In laminitis group, the dominate (>1%) phylum were *Firmicutes* (89.22%), *Actinobacteriota* (5%), *Bacteroidota* (2.01%), *Proteobacteria* (1.28%), and *Verrucomicrobiota* (1.12%).

In prophylactic group, the dominate (>1%) phylum were *Firmicutes* (64.34%), *Bacteroidota* (16.11%), *Actinobacteriota* (11.51%), *Proteobacteria* (3.75%), and *Verrucomicrobiota* (2.49%). In treatment group, the dominate (>1%) phylum were *Firmicutes* (62.65%), *Bacteroidota* (15.66%), *Verrucomicrobiota* (7.45%), *Actinobacteriota* (7.02%), and *Spirochaetota* (3.20%).

### Composition of the gut microbiota at the genus level

**Figure 10:**
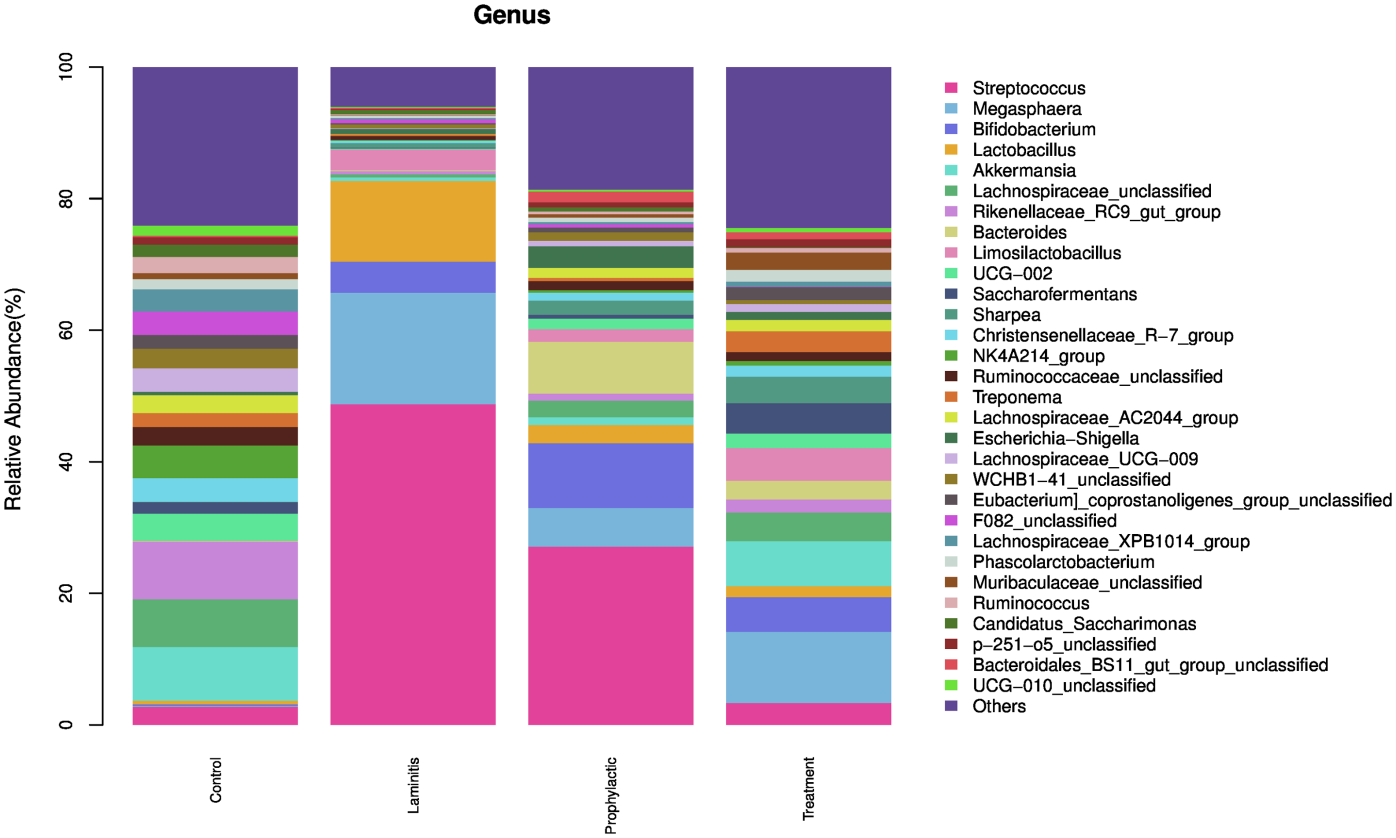
The fecal microbiota compositions in different groups at genus level.

We then analyzed the composition of the fecal microbiota, the top 5 genus in different groups (Figure 10).

In control group, the dominate (>1%) genus were *Rikenellaceae_RC9_gut_group* (8.73%), *Akkermansia* (8.17%), *Lachnospiraceae_unclassified* (7.26%), *NK4A214_group* (4.98%), *and UCG-002* (4.11%). In laminitis group, the dominate (>1%) genus were *Streptococcus* (48.75%), *Megasphaera* (16.95%), *Lactobacillus* (12.27%), *Bifidobacterium* (4.73%), and *Limosilactobacillus* (3.16%). In prophylactic group, the dominate (>1%) genus were *Streptococcus* (27.10%), *Bifidobacterium* (9.86%), *Bacteroides* (7.88%), *Megasphaera* (5.87%), and *Escherichia-Shigella* (3.31%). In treatment group, the dominate (>1%) genus were *Megasphaera* (10.83%), *Akkermansia* (6.84%), *Bifidobacterium* (5.27%), *Limosilactobacillus* (4.97%), and *Saccharofermentans* (4.62%).

We further conducted LEfSe analysis to identify the bacterial taxa that were changed in the various groups (Supplementary Figure S4). Theses taxa were spread through the four groups, specifically, 56 in control group (phyla: 1; class: 2; order: 5, family: 11, genus: 18, species: 19); 15 in laminitis group (phyla: 1; class: 1; order: 2, family: 3, genus: 3, species: 5); 36 in prophylactic group (phyla: 1; class: 1; order: 1, family: 4, genus: 12, species: 17); and, 134 in treatment group (phyla: 2; class: 8; order: 15, family: 26, genus: 42, species: 41).

The Cladogram created from LEfSe analysis showed the relationship between taxon at the levels of phylum, class, order, family, and genus (Supplementary Figure S5). Results showed that, in control group, at the genus level, the biomarkers with significant discriminative power were *Eggerthellaceae_unclassified*, *Bacteroidetes_BD2_2_unclassified*, *F082_unclassified, Prevotellaceae_UCG_004, and Rikenellaceae_RC9_gut_group*. In laminitis group, the biomarkers were *Lactobacillus, Streptococcus,* and *Megasphaera*. In prophylactic group, the biomarkers were *Bifidobacterium, Olsenella, Bacteroides, Prevotella_7,* and *Erysipelatoclostridium.* In treatment group, the biomarkers were *Muribaculaceae_unclassified, Prevotella, Prevotellaceae_UCG_001, hoa5_07d05_gut_group,* and *Parabacteroides*.

### Correlation between host clinical and serum parameters and key bacterial genera

**Figure 11:**
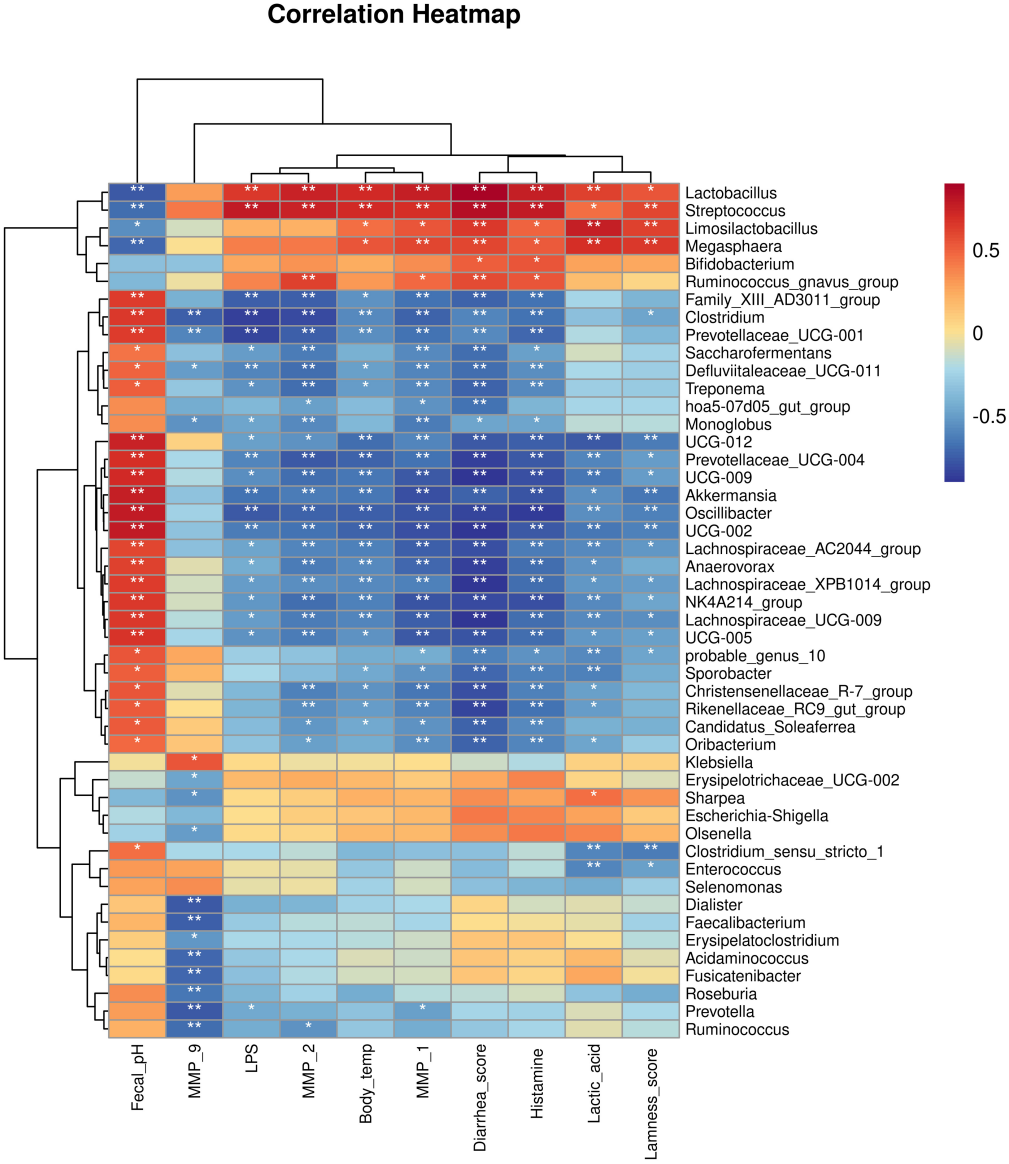
Correlation heatmap between bacterial genera and host clinical and serum parameters.

We then analyzed the relationships between host clinical and serum parameters and key bacterial genera identified by LEfSe in different groups (Figure 11). Our analysis revealed significant positive correlations between *Lactobacillus, Streptococcus, Limosilactobacillus, Megasphaera,* and *Ruminococcus_gnavus_group,* and worsened clinical and serum parameters, including elevated body temperature, diarrhea, lameness, increased inflammatory and MMPs levels, while these genera were negatively correlated with pH levels. We also noticed that there were genera negatively correlated with worsened clinical and blood parameters, including elevated body temperature, diarrhea, lameness, increased inflammatory and MMPs levels. Conversely, theses genera showed a positive correlated with pH levels.

### Predicted functional profiles of the gut microbiota community

The functional profiles of the gut bacterial community were predicted using PICRUSt2, and the results identified a total of 15 gene families in all samples at the second tier (Supplementary Figure S6). Among the predicted KEGG pathways, most of the sequences were assigned to gene families energy metabolism, environmental adaptation, endocrine system, immune system disease, immune system, xenobiotic biodegradation and metabolism, cellular processes and signaling, signal transduction, metabolism of terpenoids and polyketides, nervous system, excretory system, signaling molecules and interaction, cell mobility, and nucleotide metabolism.

**Figure 12:**
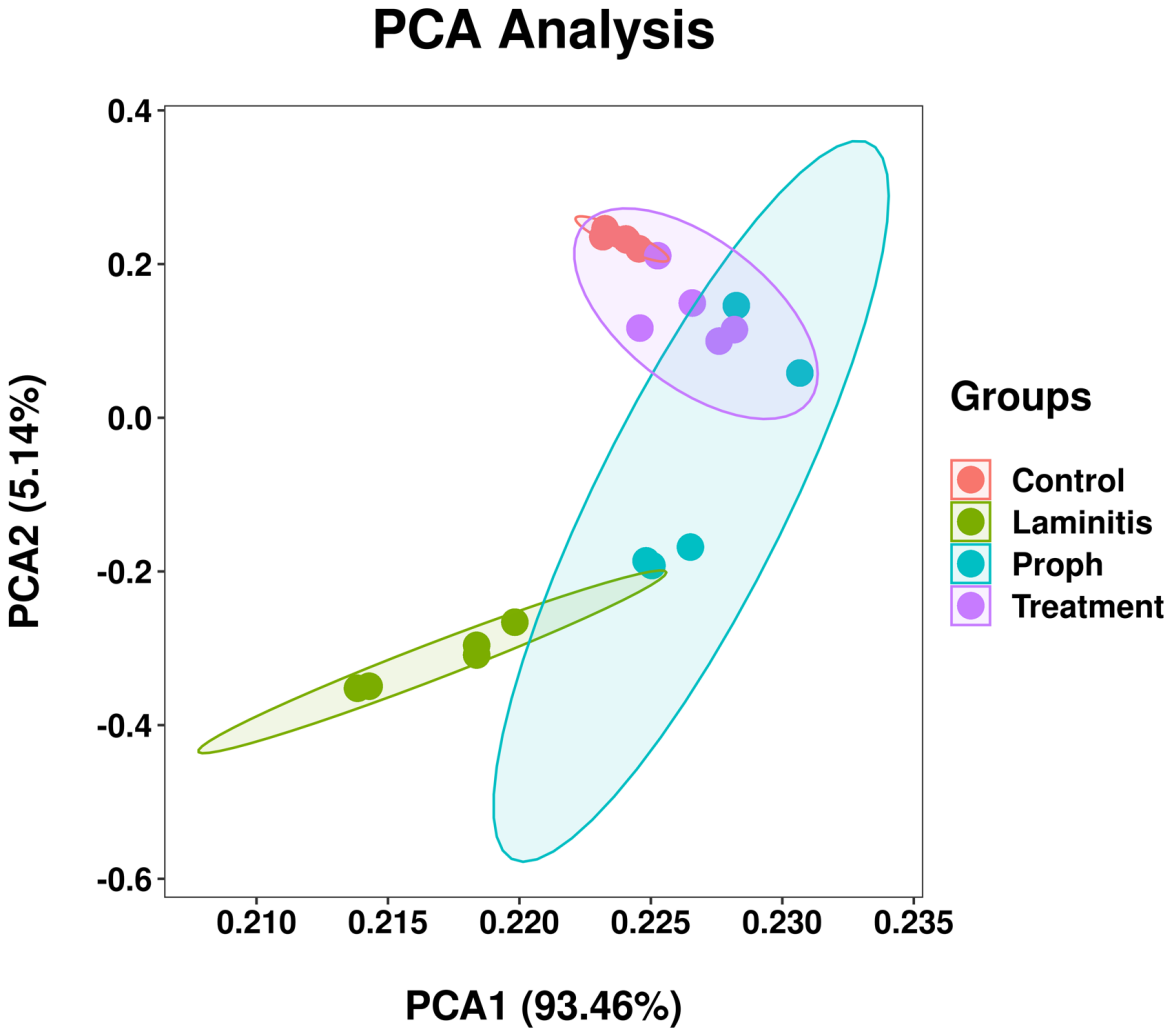
Principal component analysis (PCA) of bacterial functional diversity in different groups.

To further explore the implications of the gut bacterial functions, PCA was conducted and results showed that control group and laminitis group samples separated from each other, treatment group samples gathered together, and laminitis and prophylactic group samples gathered together. Noticeably, 2 samples from prophylactic group were closer to control group than laminitis group (*R* = 0.6563, *p* = 0.001) (Figure 12).

## Discussion

Laminitis, a condition characterized by inflammation of the hoof laminae, is a highly distressing condition that causes lameness and considerable discomfort in horses (van Eps and Pollitt, 2004; Zhang et al., 2018). In this study, we investigated the efficacy of methylated tirilazad, an anti-inflammatory agent, as a preventive and treatment option for oligofructose-induced laminitis in horses. The results showed that methylated tirilazad was effective for treating laminitis in horses.

Our study provides further evidence for a crucial link between gut microbiota dysbiosis and the development of laminitis. The observed changes in microbial composition, particularly increases in *Streptococcus*, *Lactobacillus*, *Bifidobacterium*, *Megasphaera*, and *Sharpea*, are consistent with previous findings (Tuniyazi et al., 2021). These gut microbiota alterations may trigger a cascade of events, leading to systemic inflammation and eventual laminitis. The correlation between these gut microbiota alterations and clinical symptoms highlights the potential causal relationship between gut microbiota dysbiosis and laminitis.

Oligofructose-induced equine laminitis triggered an increase in body temperature, especially at 30–32 h after model establishment, when the horse’s rectal temperature reached 39°C. Elevated body temperature has a positive effect on horses, indicating that the horse’s body begins to experience an inflammation-related response, with the immune system releasing cytokines, including interleukins and tumor necrosis factor. The horse’s intestinal and hoof lamella tissues began to show an injury response. However, a previous study reported that the body temperature of the horse would reach 38.9°C at 30–40 h after laminitis induction (Garner et al., 1975). This was inconsistent with our observation, we believe that it may be due to the fact that the horses in our present experiment were of uniform age and weight, and therefore the inflammatory response appeared at the same time, resulting in a narrower time period for the elevated body temperature to appear. Methylated tirilazad treatment significantly reduced the equine body temperature, suggesting that methylated tirilazad has an anti-inflammatory effect.

Oligofructose-induced laminitis results in a decrease in fecal pH in horses, which is consistent with our previous study (Tuniyazi et al., 2021). The direct cause of the decrease in fecal pH is acid production in the intestine. Lactic acid is the main acid produced by *Lactobacilli* and *Streptococci* in horse intestines. This phenomenon suggests that oligofructose-induced equine folliculitis may lead to a significant increase in the number of acid-producing bacteria (Tuniyazi et al., 2021). Our results showed that both methylated tirilazad treatments significantly increased the pH of equine feces, implying that this treatment may have suppressed equine intestinal *Lactobacilli* and streptococci through different mechanisms. The mechanism of action of methylated tirilazad is not yet fully understood. We hypothesize that methylated tirilazad may reduce the number of *Lactobacillus* and *Streptococcus* by indirectly acting on the intestinal flora, which in turn increases fecal pH. However, this hypothesis should be tested in future studies.

Lameness is the most typical clinical symptom of equine laminitis. Oligofructose-induced laminitis leads to apparent lameness in horses, which is due to the fact that lamella damage is the main cause of laminitis (Katz and Bailey, 2012). This suggests that oligofructose induction caused damage to the lamella, leading to the development of laminitis. The results of this study showed that methylated tirilazad treatment significantly alleviated lameness, which suggests that methylated tirilazad may restore hoof lamella damage. However, the underlying mechanisms require further investigation.

Diarrhea is a prominent symptom of dysbiosis of the intestinal flora. Consistent with previous findings (Tuniyazi et al., 2021; Milinovich et al., 2007), oligofructose-induced laminitis resulted in diarrhea in horses, suggesting that disturbances in the gut microbiota may be a key factor in the development and progression of laminitis. In our study, we found that methylated tirilazad significantly improved diarrhea symptoms in horses, implying that methylated tirilazad plays a role in regulating intestinal flora. However, it should be noted that the diarrhea index is often subjective. In addition, since these horses were young and strong, the recovery from diarrhea may not necessarily be related to methylated tirilazad administration.

Observations of hoof anatomy revealed significant visual changes. The findings showed that oligofructose-induced laminitis resulted in significant hemorrhage and swelling of the hooves. This finding further emphasizes the key role of lamellar damage in the onset and progression of equine laminitis. In our study, we found that after treatment with methylated tirilazad, the visual observation of the inside of the hoof was more similar to that of the control equine hooves, with a significant improvement in hemorrhage. This suggests that the therapeutic effect of methylated tirilazad may be related to its improvement in the blood circulation. However, the naked-eye observations did not show significant differences between the prophylactic and treatment groups.

HE and PAS staining of lamella tissues showed significant changes. Oligofructose-induced laminitis results in severe destruction of lamella tissues, and the structure is significantly damaged (Tuniyazi et al., 2021). The integrity of the lamella tissue is directly related to whether the horse develops lameness or not. The results of our study showed that after treatment with methylated tirilazad, the structure of the hoof lamella improved and showed changes that were more similar to those of the control horses. Notably, the methylated tirilazad treatment group performed significantly better than the prophylactic group, suggesting that timing may be an important factor in laminitis treatment.

Oligofructose-induced laminitis results in significantly elevated serum LPS levels in horses (Tuniyazi et al., 2021). LPS is a component of the cell wall of Gram-negative bacteria. Although endotoxin injection alone does not lead to laminitis in horses, it is vasoactive and pro-inflammatory, and is considered an important contributor to the development of laminitis (Tuniyazi et al., 2021; Reisinger et al., 2015). During episodes of carbohydrate/oligofructose overload, there is a surge in the number of gram-negative bacteria, leading to the subsequent release of LPS into the acidic gastrointestinal environment. This further leads to an increase in intestinal permeability and absorption of large amounts of LPS into the circulation, which triggers local and systemic inflammatory responses and leads to conditions such as laminitis and acidosis (van Eps and Pollitt, 2004; Zhang et al., 2018; Park et al., 2021). The methylated tirilazad intervention did not significantly change the serum levels of LPS in the prophylactic group, whereas a significant decrease in serum LPS was observed in the treatment group. This may indicate that PTP has a targeted therapeutic effect, initiating its therapeutic process only when laminitis occurs, and is accompanied by an increase in LPS. However, this hypothesis should be tested in future studies.

Oligofructose-induced laminitis results in a significant increase in serum lactate levels in horses (Tuniyazi et al., 2021). Lactate is a product of glucose metabolism under hypoxic conditions, is produced by a variety of cells and tissues (Li et al., 2022), such as skeletal muscle, and is especially pronounced during times of increased energy demand. The accumulation of lactic acid in tissues may lead to localized tissue acidosis, which in turn may trigger painful sensations (Immke and McCleskey, 2001). In addition, when horses consume carbohydrate-rich feed, the intestinal flora becomes imbalanced, leading to acidification of the intestinal environment, proliferation of lactic acid bacteria, and the conversion of acetate and format to lactate. The massive production of lactic acid in the intestinal tract subsequently enters the blood circulation through the intestinal wall, causing an increase in the blood lactate levels. In the present study, After methylated tirilazad administration, there was no significant change in the serum lactate level in the prophylactic group, whereas the serum lactate level in the treatment group was significantly reduced. This may indicate that methylated tirilazad has targeted therapeutic properties, and that its therapeutic mechanism is activated only when laminitis occurs and is accompanied by an increase in lactate levels.

Similar to previous studies on equine and bovine laminitis (Tuniyazi et al., 2021; Irwin et al., 1979), our results showed that oligofructose-induced laminitis resulted in a significant elevation of histamine levels in the serum. Histamine is a biogenic amine associated with systemic inflammation that plays an important role in allergic and inflammatory responses in the body (Maintz and Novak, 2007). Histamine may have a vasoconstrictive effect at the hoof site, reducing the blood content and flow rate in the hoof microcirculation, which leads to local tissue ischemia. Therefore, we believe that histamine is one of the causes of the destruction of hoof tissues and ultimately laminitis (Tuniyazi et al., 2021). After methylated tirilazad administration, there was no significant change in the serum levels of histamine in the prophylactic group, whereas the serum levels of histamine in the treatment group were significantly reduced. This result indicates that methylated tirilazad has a time-dependent treatment effect on laminitis, and the role of histamine in blood vessels in the pathogenesis and treatment needs further investigation.

Alterations in enzyme activity within lamella tissue were first recognized in the 1990s. Subsequent research has demonstrated that the activation of various matrix metalloproteinases, particularly MMP-2 and MMP-9, is linked to the degradation of extracellular matrix components within the lamella (French and Pollitt, 2004a, 2004b; Kyaw-Tanner et al., 2008). Changes in these enzymes lead to detachment of the basal epithelial cells of the lamella from the basement membrane, which in turn leads to the structural alterations commonly seen in laminitis. Notably, MMP-1 (also known as type I collagenase) is secreted as a preproenzyme and activated by extracellular protein hydrolysis through its regulatory peptide (Miller, 1967). Gene expression studies have demonstrated that the expression of MMP-1 is significantly upregulated in the lamella of horses with starch-induced laminitis compared to that in the control horses (Wang et al., 2014). Our results are consistent with these findings. The results showed that oligofructose-induced laminitis resulted in a significant increase in the serum levels of MMP-1, MMP-2, and MMP-9. Our study found that methylated tirilazad significantly reduced MMP-1, MMP-2, and MMP-9 levels. We believe that this may be the most significant effect of methylated tirilazad on the treatment of laminitis.

Although our study provides evidence supporting the efficacy of methylated tirilazad in the treatment of laminitis, the underlying pharmacological mechanism remains unclear. Methylated tirilazad may exert its therapeutic effects primarily through MMP inhibition and the promotion of laminar tissue repair. However, because methylated tirilazad is a novel compound, there is a limited understanding of its mechanism of action. Future studies should focus on elucidating the specific pathways through which methylated tirilazad interacts with MMPs to promote tissue repair.

Oligofructose-induced laminitis decreases the alpha diversity of equine gut microbiota (Tuniyazi et al., 2021). Alpha diversity is a key measure of species diversity and abundance in the intestinal flora (Clemente et al., 2012), which reflects the distribution and presence of different species in the intestinal microbial community under specific environmental conditions. Alpha diversity is usually measured by species richness and species evenness. Species richness refers to the number of species in the community, whereas species evenness refers to the evenness of the species distribution in the community. Maintaining balanced alpha diversity is important for the overall health of horses (Manor et al., 2020). Alpha diversity acts as an indicator of health and can be used as a metric to assess an individual’s health status. Studies have shown that alpha diversity of the gut microbiome is associated with a variety of diseases, including obesity, diabetes, cardiovascular disease, enteritis, and irritable bowel syndrome. Our results revealed that methylated tirilazad exerted a certain effect on the restoration of alpha diversity of equine intestinal flora. However, the specific mechanism by which methylated tirilazad restores the alpha diversity of equine intestinal flora has not yet been clarified, and more in-depth studies are necessary.

For beta diversity, our results showed that oligofructose-induced laminitis resulted in a significant separation compared to the control horses, suggesting differences in the composition of the gut microbiota between the two groups. The results showed that composition of the intestinal flora of horses after methylated tirilazad administration was similar to that of the laminitis group, suggesting that these treatments did not have a significant effect on the restoration of beta diversity. However, it should be noted that the recovery of intestinal microbiota takes time, and from the perspective of diarrhea relief, methylated tirilazad may have an ameliorating effect on intestinal flora.

At the phylum level, our findings revealed that the intestinal flora compositions varied among the groups, particularly *Firmicutes*, *Bacteroidota*, and *Verrucomicrobiota*. *Firmicutes* was increased 43.54% in the laminitis group compared to the control group. Following PTP-101 intervention, *Firmicutes* was reduced by 27.85 and 29.76% in the prophylactic and treatment groups, respectively, as compared to the laminitis group. *Bacteroidota* was decreased 88.93% in the laminitis group compared to the control group. After PTP-101 intervention, *Bacteroidota* was reduced by 70.15 and 67.96% in the prophylactic and treatment groups, respectively, relative to the laminitis group. *Verrucomicrobiota* was decreased 90.17% in the laminitis group compared to the control group. Interestingly, following PTP-101 intervention, *Verrucomicrobiota* levels were reduced by 22.32 and 56.43% in the prophylactic and treatment groups, respectively, as compared to the laminitis group. These three phyla account for more than 91% of the total population, indicating their significant role in the development and recovery of laminitis. Therefore, targeting these phyla may offer a potential therapeutic approach for gut microbiota dysbiosis-induced laminitis in horses.

Consistent with earlier studies (Tuniyazi et al., 2021), our findings showed that oligofructose-induced laminitis resulted in an increase in the genera *Streptococcus*, *Lactobacillus*, *Bifidobacterium*, *Megasphaera*, and *Sharpea*, which is in line with previous research. However, our study also revealed a difference in the increase of *Limosilactobacillus*, which differs from previous findings of an increase in *Sharpea*. This discrepancy may be attributed to differences in the horses used in the study, such as age and living environment, or to the fact that the sequencing results were based on pre-existing DNA sequences, which could have led to differences in the sequencing results as more data became available. Nonetheless, the primary bacterial genera responsible for equine laminitis remain consistent.

*Streptococcus* is a Gram-positive coccobacillus that is widely found on mucosal surfaces in animals and humans, including in the intestinal tract of horses (Tuniyazi et al., 2021). *Streptococci* may play important ecological and pathogenic roles in animal guts. *Streptococci* may be involved in the development and progression of laminitis in several ways, including adhesion and invasion, inflammation mediation, toxin production, and immunomodulation. *Streptococci* may enter circulation and ultimately affect the lamella through adhesion and invasion of the intestinal mucosa. This may lead to the activation of the immune response and inflammation. *Streptococci* may trigger an inflammatory response in the immune system. Inflammation is a key component in the development of laminitis and can lead to symptoms, such as tissue damage, vasodilatation, and pain. It may produce a number of toxins, such as hemolysins, which may cause direct damage to lamella tissue and stimulate an immune response.

As a key member of the gut microflora, *Lactobacillus* positively affects host health (Pessione, 2012). It plays an important role in several ways, including the maintenance of intestinal microecological balance, production of lactic acid and other metabolites with inhibitory effects, enhancement of intestinal barrier function, modulation of the immune system, participation in nutrient metabolism, and reduction of intestinal pH. However, when oligofructose induces laminitis, lactic acid bacteria proliferate and produce lactic acid. It has been found that oligofructose, a soluble dietary fiber, is difficult to digest in the intestinal tract and therefore produces large amounts of lactic acid through fermentation by intestinal bacteria such as *Lactobacilli* (Tuniyazi et al., 2021). Excessive accumulation of lactic acid may lead to acidification of the intestinal environment, which in turn disrupts the normal intestinal microbial community balance and contributes to the development of intestinal inflammatory diseases, such as acidosis and laminitis. In addition, the overproduction of lactic acid may adversely affect the host immune system and intestinal mucosa.

The acidic environment may affect the intestinal barrier function, making the intestinal mucosa susceptible to damage. Additionally, lactic acid may affect the activity and function of immune cells and disturb the balance of immune regulation. These changes may lead to further exacerbation of intestinal inflammation and disorganization of the bacterial communities, thus promoting the development of laminitis. In summary, the proliferation of lactic acid bacteria and the excessive accumulation of lactic acid during oligofructose-induced laminitis may adversely affect the balance of the intestinal microbial community, intestinal mucosa, and immune system, thus promoting the occurrence and development of laminitis.

Detailed information on the specific role of *Bifidobacteria* in the development and progression of laminitis is lacking and requires further in-depth investigation. However, it is understood that *Bifidobacteria* are a group of Gram-positive, non-motile, strictly anaerobic, rod-shaped bacteria that are widely found in habitats such as the gastrointestinal tract, vagina, and oral cavity of humans and animals. Bifidobacteria can produce short-chain fatty acids (SCFAs), such as lactic acid and acetic acid through fermentation, which are important for gut health and immune function. We believe that the ability to produce acid may be a key factor in its involvement in laminitis.

Regarding the specific role of *Megasphaera* in oligofructose-induced laminitis, although there are still many aspects that need to be further investigated in depth, based on the results of the available studies, we can speculate on the possible mechanisms of action. First, *Megasphaera* have the property of fermenting acid-producing metabolites, and during oligofructose-induced intestinal flora, *Megasphaera* may produce lactic acid and propionic acid through fermentation of oligofructose, which may lead to acidification of the intestinal environment. Second, the over proliferation and acid-producing effects of lactic acid bacteria such as *Megasphaera* may lead to acidification of the intestinal internal environment, which may alter the balance of the intestinal microbiome, affect the growth and activity of other flora, and induce an intestinal inflammatory response. In addition, increased acid-producing bacteria, especially excessive acid production by bacteria such as *Megasphaera*, may increase acidity of the internal environment, resulting in damage to the intestinal mucosal barrier. This may lead to the entry of bacteria and their metabolites into the intestinal wall through the mucosal barrier, stimulating an inflammatory response and damaging the host tissue. Therefore, an in-depth study of the specific role of *Megasphaera* in equine oligofructose-induced laminitis will help us better understand the mechanism of laminitis and provide an important basis for treatment and prevention.

There is a lack of systematic research and literature on the specific role of bacteria of the genus *Sharpea* in oligofructose-induced laminitis. The role and function of bacteria of this genus in the equine intestinal flora need to be further elucidated. Similarly, the role and function of *Sharpeain* strains in the equine intestinal flora have not been extensively studied and understood. In particular, there is very limited information available on *Sharpea*’s specific roles. Bacteria in oligofructose-induced laminitis. Therefore, more research and literature support are needed to gain insight into the specific role and effects of bacteria of the genus *Sharpeain*.

In summary, these results suggest that a normal gut microbiota composition is essential for equine health. Therefore, regulating the intestinal flora and reducing the relative abundance of these genera through targeted modulation may be key to alleviating the symptoms of equine laminitis and a viable approach to treating and preventing laminitis in horses. Our results showed that methylated tirilazad reduced the relative abundances of *Streptococcus*, *Lactobacillus*, *Bifidobacterium*, *Megasphaera*, and *Limosilactobacillus*. These results suggest that methylated tirilazad affects the intestinal flora. LEfSe and classification tree analyses further validated these results, that is, the relative abundances of *Streptococcus*, *Lactobacillus*, *Megasphaera*, and *Limosilactobacillus* were significantly lower in the prophylactic group, whereas the relative abundance of *Limosilactobacillus* was significantly lower in the treatment group. In addition, Pearson correlation analysis further verified that equine intestinal flora may play an important role in the development and recovery of laminitis. The results showed that the genera directly associated with the development of laminitis were positively correlated with the worsening of clinical signs and blood markers, which could help further our understanding of the relationship between laminitis and gut microbiota. Pearson correlation analysis could also help us to screen for probiotics for the treatment of laminitis, but further research is needed to investigate the specifics of this. Finally, we noted changes in the predicted function of the flora in horses with laminitis, with different treatments altering the function of the flora. This suggests that, in addition to the microbiota itself, bacterial function and metabolites play a role in the development, progression, and recovery of laminitis. Previous studies have shown that the development of laminitis is closely related to intestinal metabolites (Costa et al., 2012), but the specific role of intestinal metabolites in different treatments still requires further investigation.

The potential of combining methylated tirilazad with other therapeutic approaches warrants further investigation. In an ongoing study on the use of fecal microbiota transplantation in conjunction with methylated tirilazad, we obtained promising preliminary results. This combination therapy approach could potentially address both systemic inflammation and gut dysbiosis aspects of laminitis. Future research should consider the adjunct use of probiotics with methylated tirilazad to further modulate gut microbiota and enhance treatment outcomes.

However, there are several limitations of our study that need to be addressed. First, the composition of the equine gut microbiota is highly individualized and can be influenced by factors such as diet, age and breed (Garber et al., 2020). Although we attempted to mitigate these effects by using horses of the same age and breed, it is important to consider that certain breeds may be more susceptible to laminitis. Therefore, future studies should include different breeds of horses to fully investigate the effects of methylated tirilazad. In addition, our study lacked long-term follow-up and therefore could not assess the sustained clinical improvement or potential delayed side effects of methylated tirilazad intervention. To address this limitation, future studies should extend the duration of the study to carefully evaluate the long-term effects of methylated tirilazad and monitor for any side effects that may occur over time. Notably, resource limitations prevented the analysis of pro-inflammatory cytokines in this study. Future research should include assessment of these markers to provide a more comprehensive picture of the inflammatory response in laminitis. Finally, our study did not include tissue-level analysis of MMP expression, which could have provided more direct evidence of the effect of methylated tirilazad on laminar tissue. Future studies should incorporate this tissue-level analysis to clarify the local effects of methylated tirilazad on hoof laminae.

## Conclusion

This study demonstrates that methylated tirilazad, a novel anti-inflammatory agent, shows substantial promise for the treatment of equine laminitis. Considering its reduction in MMP activity, alleviation of clinical symptoms, and partial modulation of the gut microbiota, methylated tirilazad represents a new approach for laminitis management. Although the underlying mechanism of action requires further investigation, the observed improvements in lameness scores and laminar tissue structure highlight the potential of methylated tirilazad to serve as a valuable addition to the limited array of available laminitis treatments. Future long-term studies across diverse horse populations, along with more detailed mechanistic investigations, are needed to fully establish the role of methylated tirilazad in laminitis therapy and potentially transform the current management approach to this serious condition.

## Data Availability

The 16S rRNA data is uploaded in NCBI SRA with accession number: PRJNA1080359. The remaining datasets presented in this study can be& found in online repositories. The names of the repository/repositories and accession number(s) can be found in the article/Supplementary material.

## Footnotes

^1^ https://github.com/murdoch103/random_horse.git

^2^ https://www.omicstudio.cn/

## Supplementary material

SUPPLEMENTARY FIGURE S1 The preparing process of methylated tirilazad for equine IV administration.

SUPPLEMENTARY FIGURE S2 Alpha diversity of gut microbiota in different groups of horses. **(A)** Observed species. **(B)** Shannon. **(C)**, Simpson. **(D)** Chao1. **(E)** Goods coverage. **(F)** Pielou_e.

SUPPLEMENTARY FIGURE S3 Beta diversity of gut microbiota in different groups of horses. **(A)** PCA plot. **(B)** PCoA plot. **(C)** UGMA cluster plot. **(D)** NMDS plot.

SUPPLEMENTARY FIGURE S4 LEfSe analysis showing the relationship between taxon at the levels of phylum, class, order, family, genus, and species.

SUPPLEMENTARY FIGURE S5 Cladogram generated from LEfSe analysis showing the relationship between taxon at the levels of phylum, class, order, family, genus, and species.

SUPPLEMENTARY FIGURE S6 PICRUSt2 plot of different groups.
