# Supplementary figures and images for: Methylated tirilazad may mitigate oligofructose-induced laminitis in horses
Source: Front Microbiol. 2024 Sep 25;15:1391892. doi: 10.3389/fmicb.2024.1391892 (PMC11461245; doi:10.3389/fmicb.2024.1391892)

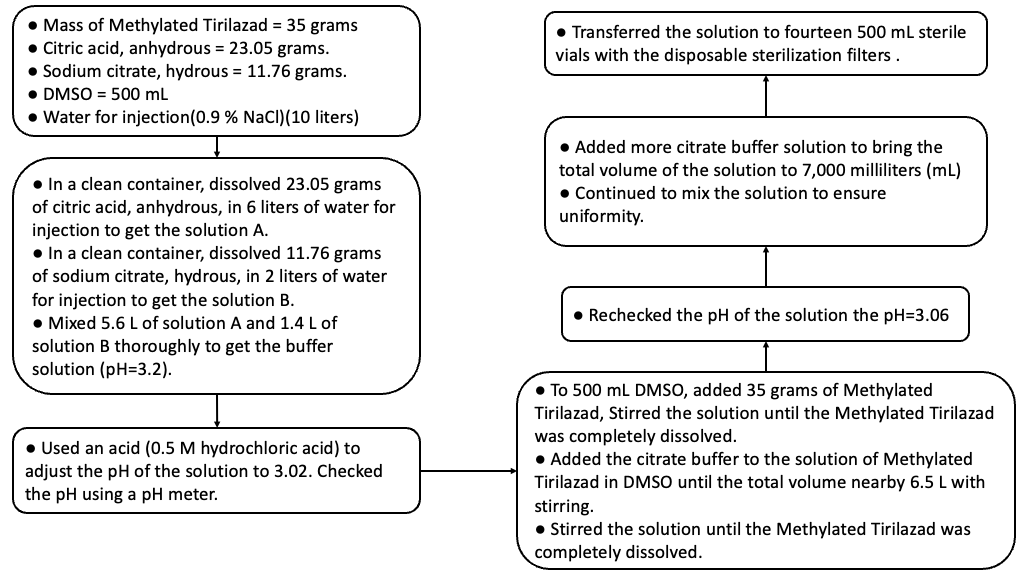

Supplement: SUPPLEMENTARY FIGURE S1 — The preparing process of methylated tirilazad for equine IV administration. [file Image_1.TIFF]

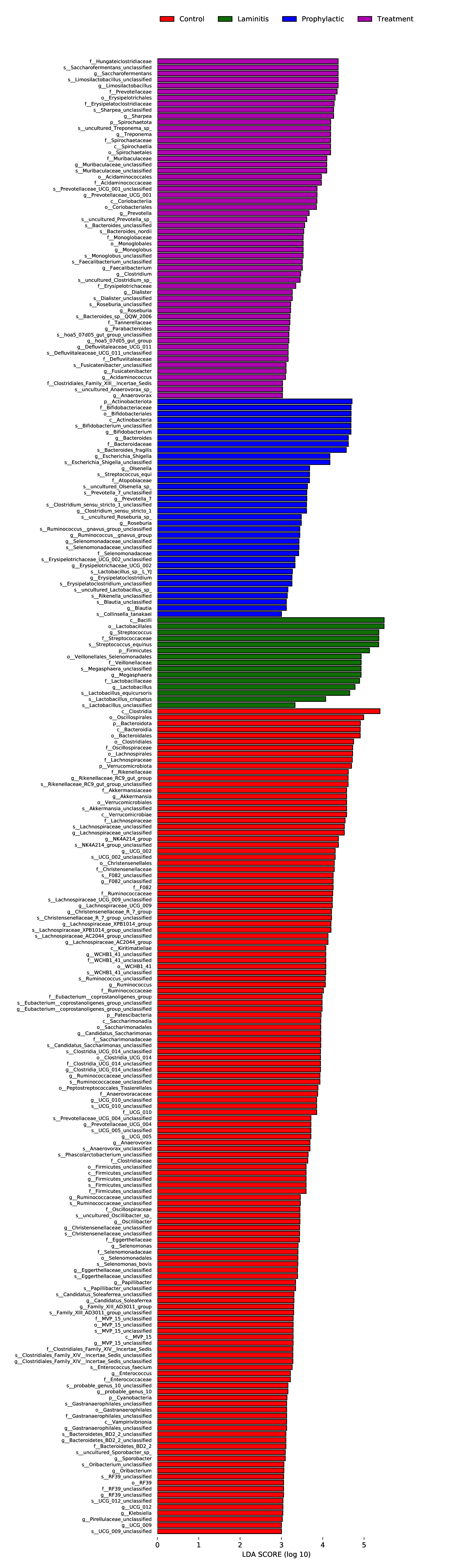

Supplement: SUPPLEMENTARY FIGURE S4 — LEfSe analysis showing the relationship between taxon at the levels of phylum, class, order, family, genus, and species. [file Image_4.TIFF]

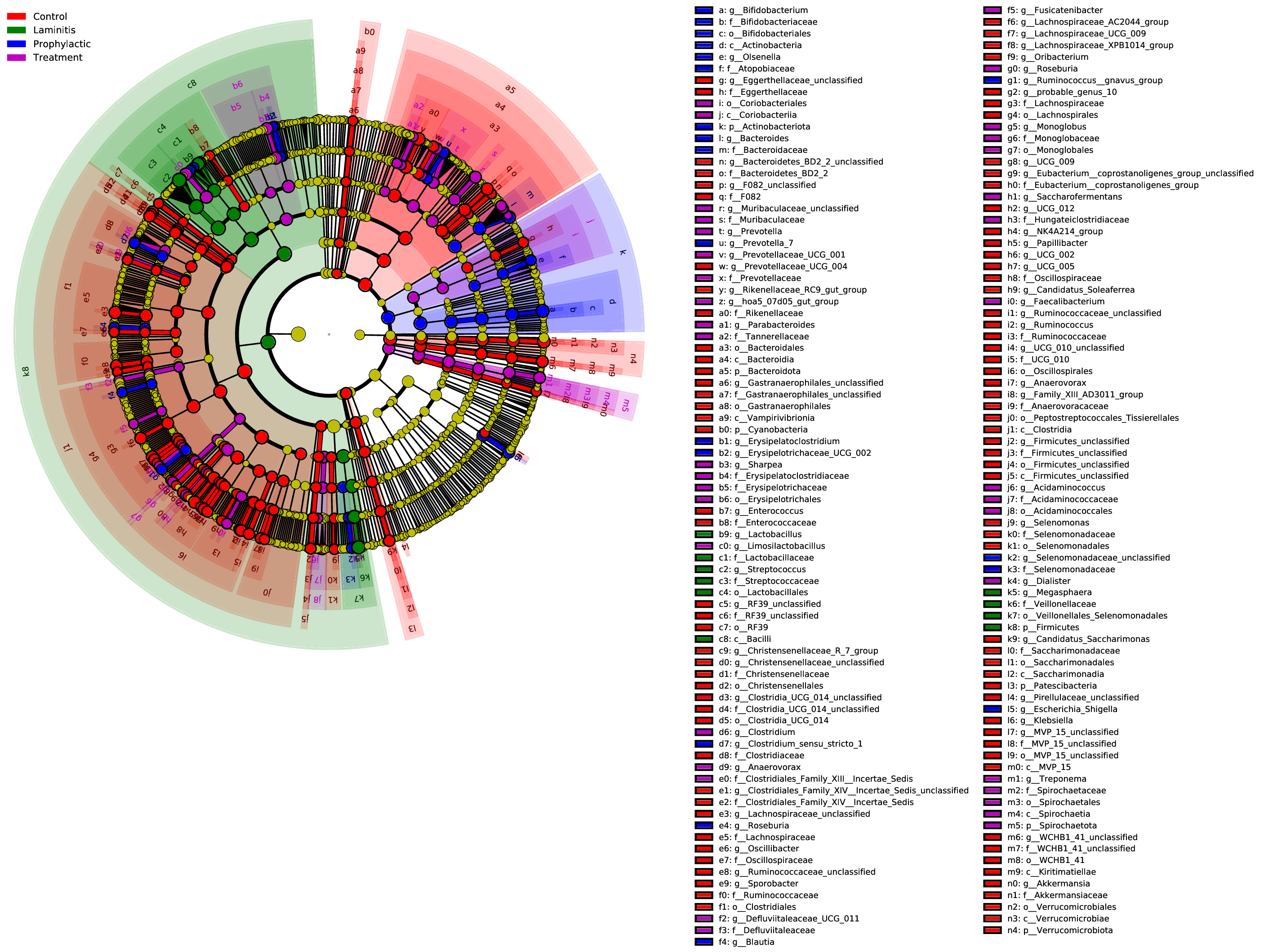

Supplement: SUPPLEMENTARY FIGURE S5 — Cladogram generated from LEfSe analysis showing the relationship between taxon at the levels of phylum, class, order, family, genus, and species. [file Image_5.TIFF]

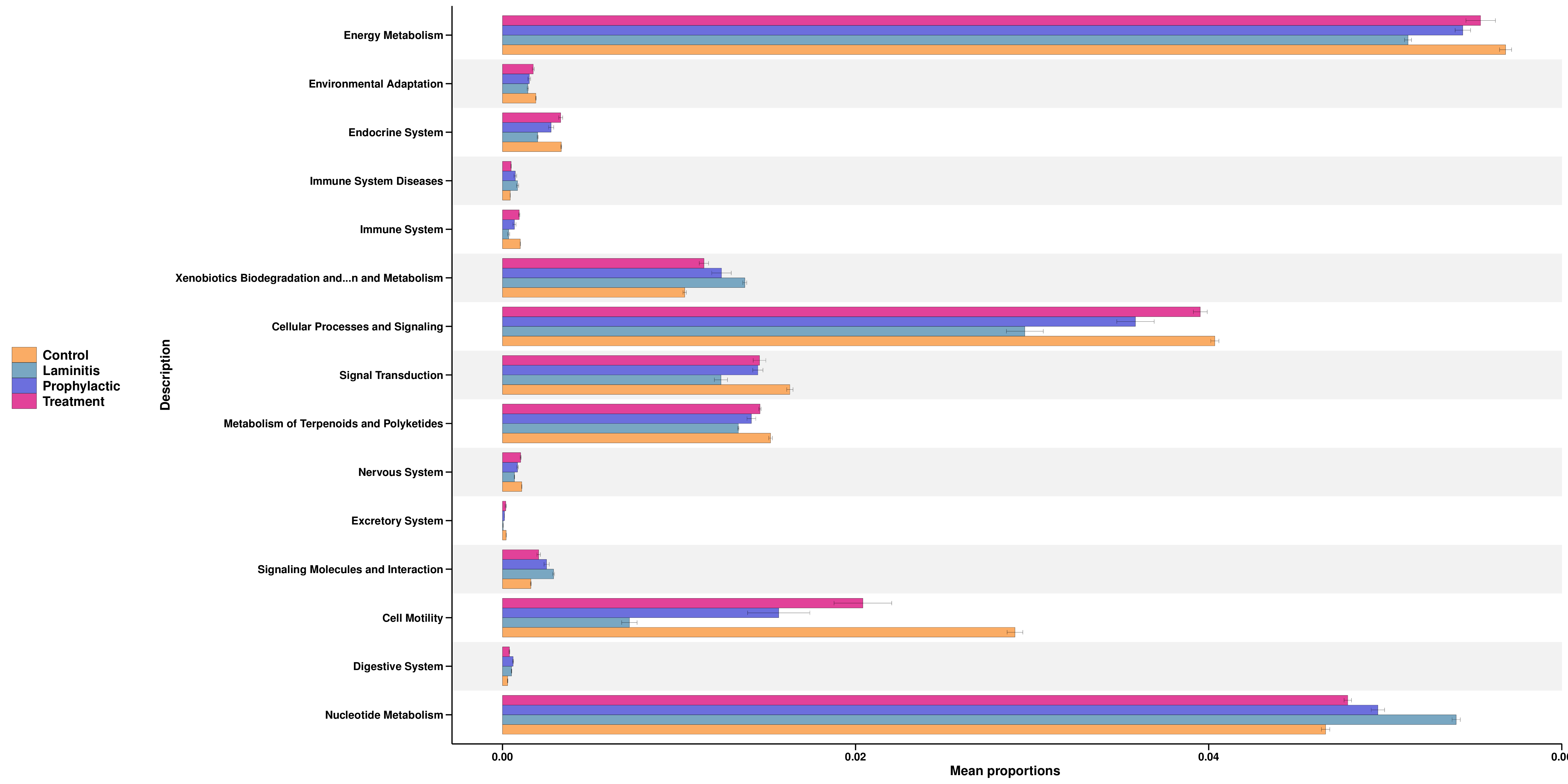

Supplement: SUPPLEMENTARY FIGURE S6 — PICRUSt2 plot of different groups. [file Image_6.TIFF]
